# Genomic characterization of arboviruses discovered between the 1950s and 1980s

**DOI:** 10.1128/jvi.01214-25

**Published:** 2025-09-08

**Authors:** Ingra M. Claro, Xinyi Hua, Ashley Viveros, Filipe R. R. Moreira, José Luiz Proença-Módena, Kenneth S. Plante, Scott C. Weaver, William M. de Souza

**Affiliations:** 1 Department of Microbiology, Immunology and Molecular Genetics, College of Medicine, University of Kentucky, Lexington, Kentucky, USA; 2 Department of Microbiology and Immunology, University of Texas Medical Branch 12338, Galveston, Texas, USA; 3 Department of Genetics, Institute of Biology, Federal University of Rio de Janeiro 28125, Rio de Janeiro, Brazil; 4 Laboratory of Emerging Viruses, Department of Genetics, Microbiology and Immunology, Institute of Biology, University of Campinas 124594, Campinas, Brazil; 5 World Reference Center for Emerging Viruses and Arboviruses, University of Texas Medical Branch 12338, Galveston, Texas, USA; 6 Global Virus Network, Baltimore, Maryland, USA; St. Jude Children's Research Hospital, Memphis, Tennessee, USA

**Keywords:** arboviruses, vector-borne diseases, genomic characterization, orbivirus, orthobunyavirus, rhabdovirus, alphavirus, flavivirus

## Abstract

Arthropod-borne viruses (arboviruses) pose a major threat to global public health, impacting both human and animal health. Genomic characterization is important for arboviruses because it allows for an understanding of their evolution and improves timely outbreak and epidemic response. In this study, we used high-throughput sequencing and computational analyses to characterize the genomes and evolution of 46 previously unsequenced or partially sequenced arbovirus isolates collected across 23 countries between 1954 and 1984. Our analyses revealed that these viruses belong to 11 genera in eight viral families, including *Sedoreoviridae* (*n* = 26), *Peribunyaviridae* (*n* = 12), *Rhabdoviridae* (*n* = 3), *Spinareoviridae* (*n* = 1), *Phenuiviridae* (*n*=1), *Togaviridae* (*n*=1), *Flaviviridae* (*n*=1), and *Nairoviridae* (*n* = 1). Based on our genomic and evolutionary characterization, we propose 15 novel arbovirus species, including six that are likely mosquito-borne, six likely tick-borne, and three isolated from birds, lizards, and humans. Furthermore, we found that the Ossa virus (genus *Orthobunyavirus*) is a reassortant, possessing a unique M segment and S and L segments derived from the Madrid virus (*Orthobunyavirus madridense*), while the Brus Laguna virus (*Orthobunyavirus gamboaense*) exhibits an M segment derived from the Alajuela virus (*Orthobunyavirus gamboaense*). Additionally, our viral genome composition-based analysis indicates that Lanjan, Zingilamo, Tindholmur (*Orbivirus magninsulae*), and Bauline viruses (*Orbivirus magninsulae*) have a high likelihood of producing human infection, suggesting the risk of emergence and indicating the need for further experimental investigation. Collectively, our findings contribute to the understanding of arbovirus diversity, evolution, and taxonomy, providing a valuable genomic resource for future research on arbovirus biology and emergence potential.

**IMPORTANCE:**

Genomic data can reveal critical insights into virus ecology and evolution and become critical to viral surveillance systems. In the last decades, advancements in genomic technology have significantly expanded the discovery of new viruses. However, many arthropod-borne viruses (arboviruses) isolated before the genomic era lack sequencing information, which limits our understanding of their impact on human and animal health. In this study, we sequenced 46 previously unsequenced or partially sequenced arbovirus isolates collected from 23 countries between the 1950s and 1980s. Next, we conducted a comprehensive genomic and evolutionary characterization, which revealed that these viruses belong to 11 genera across eight viral families. Our findings expand the genomic data of arboviruses, which can be used for the development of detection methods and contribute to a better understanding of their diversity, evolution, taxonomy, and pathogenic potential.

## INTRODUCTION

Arthropod-borne viruses (arboviruses) pose a significant and growing threat to global public health, causing substantial human and animal morbidity and mortality (1, 2). These viruses are transmitted by hematophagous arthropods to vertebrate hosts, including humans and domestic animals (2). Arboviral infections often cause undifferentiated febrile illness in humans, but they can also lead to severe syndromes such as neurological disease, hemorrhagic fever, chronic polyarthritis, abortions, and congenital disorders (1–3). Similarly, arboviruses can also cause neurological diseases, abortions, and congenital disorders in livestock (4). Furthermore, arboviral diseases disproportionately affect the most vulnerable populations, causing hundreds of millions of cases annually, often overwhelming healthcare systems and leading to substantial economic burdens across numerous countries (5). At present, most arboviral diseases lack effective preventive vaccines or specific antiviral treatments (6).

The most significant human arbovirus in terms of global disease burden is the dengue virus, which has expanded its geographical transmission range since the 1950s, causing approximately 96 million cases and 40,000 deaths annually; over 3.9 billion people in 129 countries are at risk of infection (7, 8). In addition, several arboviruses have recently emerged or re-emerged, causing widespread outbreaks in humans and/or domesticated animals, such as West Nile, chikungunya, Zika, bluetongue, African swine fever, western equine encephalitis, and Oropouche viruses (9–18). Furthermore, arboviruses can cause significant ecological disruption after their introduction. For instance, WNV has a significant negative impact on North American avian populations, while YFV poses a major threat to non-human primates in South America (19–21).

Predicting and anticipating the emergence and re-emergence of arboviruses remains challenging, with multiple factors contributing to these events, including unplanned urbanization, increased human mobility, uncontrolled vector expansion, forest degradation, and climate change (2, 22). Therefore, strengthening preparedness for emerging arboviral diseases is critical for the early identification of outbreaks, preventing their spread, and developing effective strategies to mitigate and reduce their burden at the human-animal-ecosystem interface (23–25). Rapid characterization of etiological agents during pathogen emergence plays a pivotal role because it allows for the timely development of diagnostic methods, infers evolutionary relationships and pathogenic potential, and informs evidence-based public health and medical decision-making (13, 25). However, genomic information is lacking for many arboviruses discovered before 2003, when the genomic era began (26). To fill this gap, we used high-throughput sequencing and computational biology to sequence and characterize previously partially sequenced and unsequenced arboviruses discovered between the 1950s and 1980s.

## MATERIALS AND METHODS

### Virus isolates and RNA extraction

Virus isolates were obtained from the World Reference Center for Emerging Viruses and Arboviruses at the University of Texas Medical Branch in Galveston, Texas, USA. Viral RNA was extracted directly from either homogenized infected suckling mouse brain tissue or culture supernatants of BHK-BSR, C6/36, or Vero cells (Table S1). Then, viral RNA was extracted using the QIAamp Viral RNA Mini Kit (Cat no. 52904, Qiagen, USA), and residual DNA was removed using TURBO DNase (Cat no. AM2238, Thermo Fisher Scientific, USA). RNA quantification was performed using the Qubit RNA HS Assay Kit (Cat no. Q10211, Thermo Fisher Scientific, USA) and Qubit 3.0 Fluorometer (Invitrogen, USA). RNA integrity and purity were measured using an Agilent 2100 Bioanalyzer (Agilent Technologies, USA).

### Viral sequencing and genome assembly

RNA-seq libraries were prepared using the NEBNext Ultra II RNA Library Prep Kit (Cat no. E7770L, New England Biolabs, USA) for Illumina, incorporating the NEBNext rRNA Depletion Kit (Cat no. E6310X, New England Biolabs, USA) according to the manufacturer’s instructions. Libraries were pooled at equimolar concentrations and sequenced on an Illumina NextSeq 2000 instrument (Illumina, USA) using paired-end, 150-base reads. Sequencing reads were then trimmed to remove Illumina adapter sequences and low-quality bases. Next, reads were assembled *de novo* using Abyss version 2.3.7 (27). Contigs were subsequently clustered and subjected to BLAST searches against the NCBI viral protein and nucleotide sequence databases. Identified viral contigs were extended using Jellyfish version 2.3.0 and scaffolded into full-length genomes (28).

### Viral genomic characterization

Putative open reading frames (ORFs) and their corresponding amino acid sequences were predicted using Geneious Prime version 2024.0.5 (Biomatters, New Zealand) and BLASTX searches. Signal peptides and their cleavage sites were predicted using SignalP version 6.0 (29). Potential motifs characteristic of viral families were detected using Geneious Prime version 2024.0.5. Multiple sequence alignments (MSAs) of nucleotide and amino acid sequences were performed using MAFFT version 7.525 (30) and manually curated using AliView version 1.28 (31).

### Phylogenetic analysis, genetic distances, and reassortment analysis

Maximum likelihood (ML) phylogenetic trees were generated using MSA of complete amino acid sequences of arboviruses sequenced in this study, alongside representative sequences from relevant viral genera or families, based on the International Committee on Taxonomy of Viruses (ICTV) taxonomy (32). ML trees were inferred using the IQ-TREE version 2.1.4-beta, employing the best protein substitution model determined by modelFinder (33). Node support was evaluated with 1,000 ultrafast bootstrap replicates (34). Phylogenetic trees were visualized using FigTree version 1.4.4. Genetic distance was calculated on Geneious Prime v.2024.0.5, and reassortment events in segmented arboviruses were screened with RDP version 5 (35).

### Emergence risk analysis from viral genomes

The potential emergence risk and likelihood of infection in humans were based on viral nucleotide genomes and performed by using the genome composition-based model as previously described (36). Features analyzed include codon usage biases, amino acid composition, dinucleotide biases, and sequence similarity to human RNA transcripts. Emergence risk probabilities were categorized into four tiers—low, medium, high, and very high based on a cutoff value of 0.293, which was previously determined by a balance of sensitivity and specificity during model development (36). The classification criteria were determined by the overlap of predicted probability confidence intervals. Low: entire 95% confidence interval (CI) of predicted probability equal to or greater than cutoff; medium: mean prediction equal to or greater than cutoff, but CI crosses it; high: mean prediction higher than cutoff, but CI crosses it; very high: entire CI higher than cutoff.

## RESULTS

### Characteristics of historical arbovirus isolates

**Fig 1:**
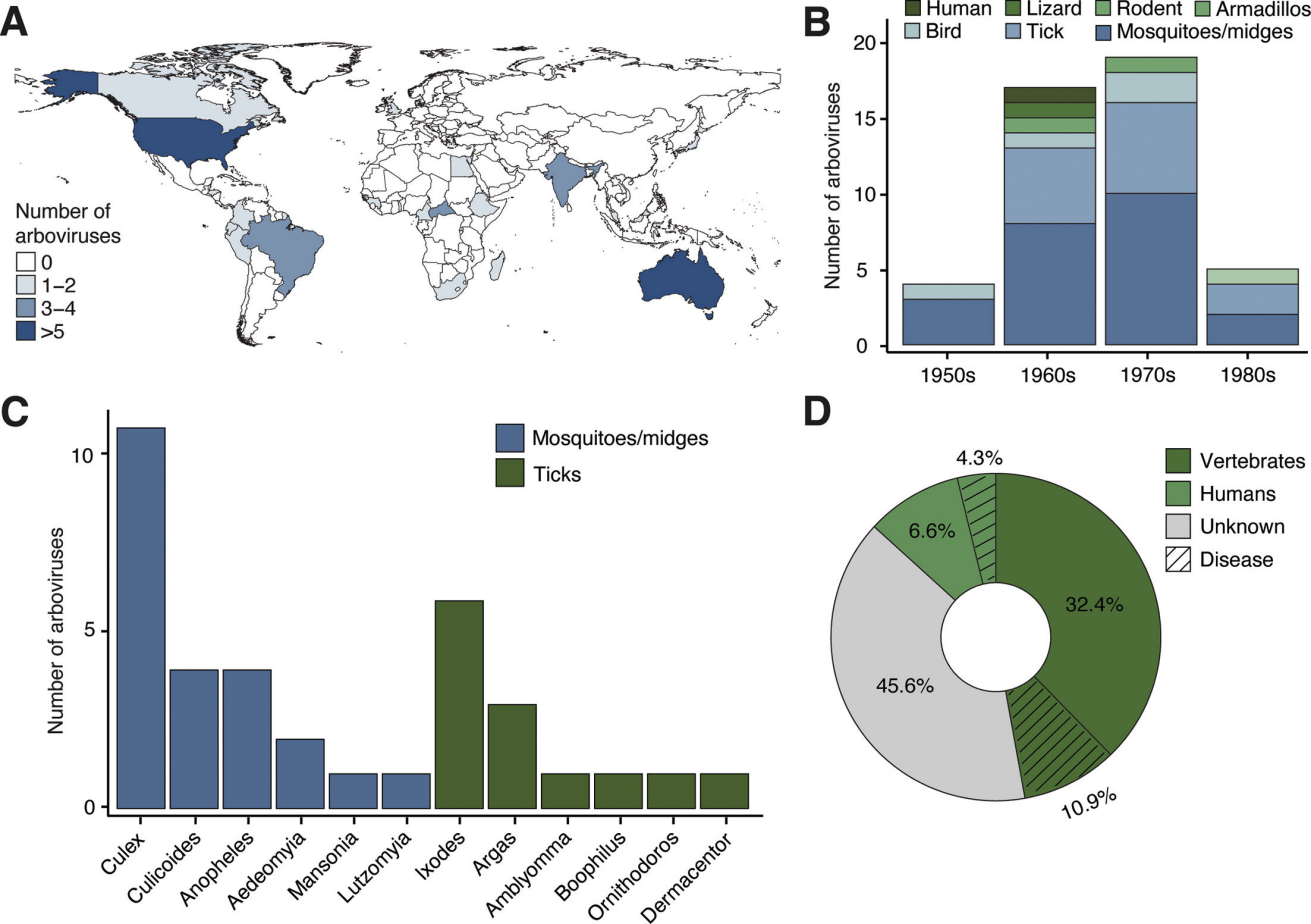
Characteristics of sequenced arbovirus isolates. (**A**) Map colored according to the geographic origin of arbovirus isolates sequenced in this study. Map was generated with RStudio. (**B**) Distribution of the number of arbovirus isolates sequenced in this study by decade, categorized by the discovered host. (**C**) Number of arbovirus isolates sequenced in this study from arthropod hosts, categorized by genus. (**D**) Percentage of arboviruses in this study recognized to cause infection and/or disease in humans and other vertebrates.

We sequenced and characterized the genomes of 46 distinct arboviruses discovered across 23 countries between 1954 and 1984, which were not previously fully sequenced and were available at the World Reference Center for Emerging Viruses and Arboviruses (Fig. 1A and B). The majority of arboviruses were identified in samples from Australia (17.4%, 8 of 46), followed by the United States of America (13%, 6 of 46). Most isolates were collected during the 1960s (37.0%, 17 of 46) and 1970s (41.3%, 19 of 46) (Fig. 1B; Table S1). The arbovirus isolates were originally obtained from vertebrates (reptiles and mammals, including humans) and arthropods, which accounted for 78.3% (36 of 46) (Fig. 1B and C). Among the arthropod hosts, mosquitoes were the predominant source (63.9%, 23 of 36), primarily the *Culex* genus (47.8%, 11 of 23), followed by *Anopheles* mosquitoes and *Culicoides* midges (17.4%, 4 of 23 each). Arboviruses identified in ticks corresponded to 36.1% (13 of 36), with the *Ixodes* genus representing 46.2% (6 of 13) (Fig. 1C; Table S1). The arbovirus isolates had diverse passage histories up to 30 times, typically propagated in Vero (African green monkey) or C6/36 (*Aedes albopictus*) cell cultures. Detailed passage information is provided in Table S1. We found that 43.5% (20 of 46) were known to infect vertebrates (non-humans), including 10.9% (5 of 46) that cause veterinary diseases. A total of 10.9% (5 of 46) cause human infections, with 4.3% (2 of 46) causing disease in humans. Additionally, 45.6% (21 of 46) remain unknown regarding vertebrate and human infections (Fig. 1D).

### Genomic sequencing of historical arbovirus isolates revealed discrepancies with serological classifications

**Fig 2:**
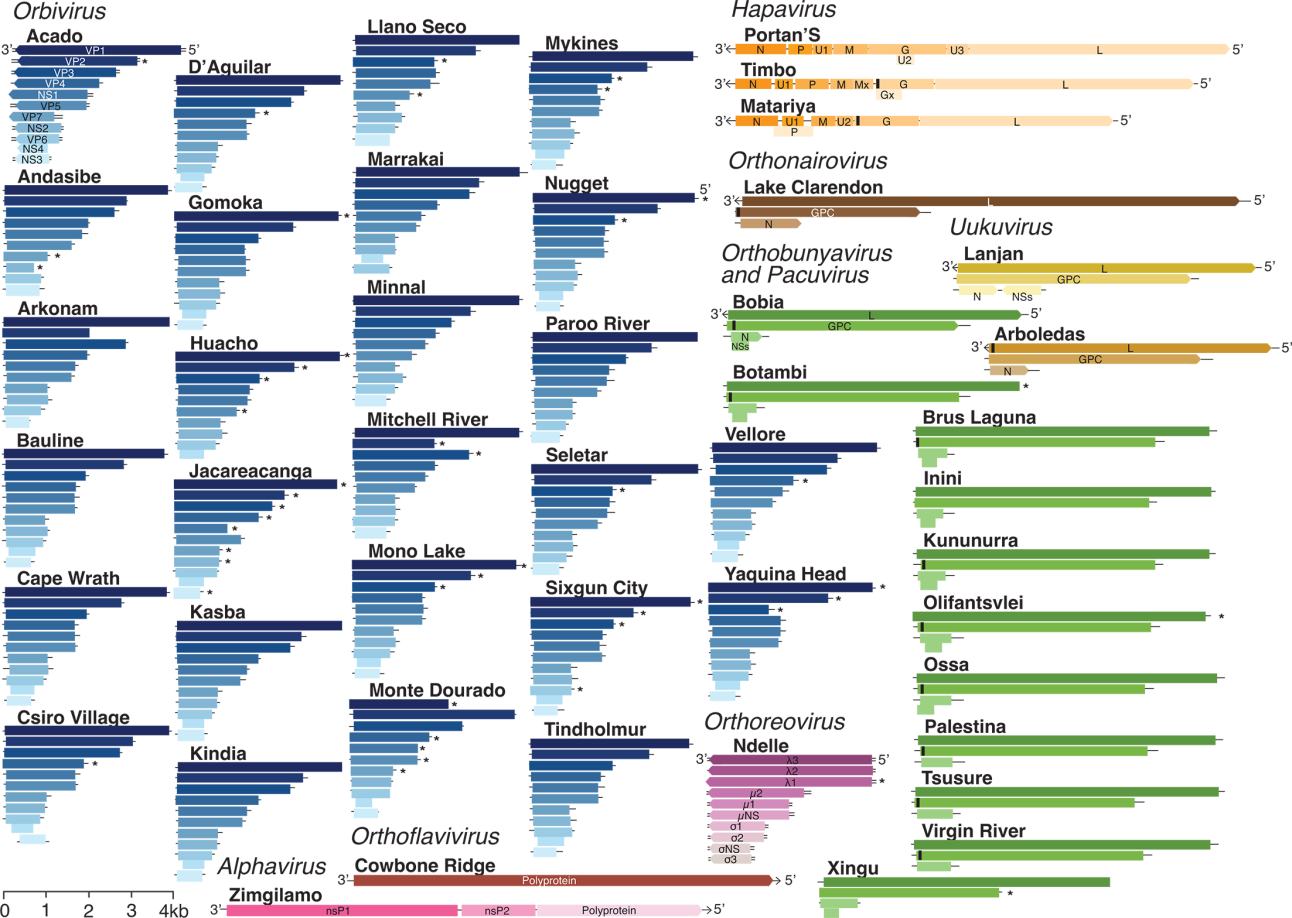
Genome organizations of historical arbovirus isolates characterized in this study. These include segments, genome length, encoded proteins, and genome polarity. The length of genomes is indicated by the scale. Black bars represent signal peptide locations (Table S3). Asterisks indicate viruses with incomplete open reading frame sequences. Segment names are shown for the first virus listed in each genus, with consistent colors applied to viruses within the same genus. VP, viral protein; Ns/nsP, non-structural proteins; RdRp, RNA-dependent RNA polymerase; L, large segment; GPC/G, glycoprotein; N, nucleoprotein; P, phosphoprotein; M, matrix protein; kb, kilobases.

We generated near-complete ORF sequences for all 46 arboviruses, with depths of 9.7 to 253.8 (Table S2). Of these, 56.5% (26 of 46) represented previously unsequenced viruses, 28.3% (13 of 46) had partial genome sequences available, including 11 from the same strain, and for 15.2% (7 of 46), nearly complete coding sequences became available during this study (Table S2). Our similarity analysis at the amino acid level, based on arbovirus genomes analyzed using BLAST, revealed that the 46 arboviruses belonged to eight distinct viral families across 11 genera. These families were *Sedoreoviridae* (genus *Orbivirus*, *n* = 26), *Spinareoviridae* (genus *Orthoreovirus*, *n* = 1), *Peribunyaviridae* (genera *Orthobunyavirus* and *Pacuvirus*, *n* = 12), *Rhabdoviridae* (genera *Hapavirus*, *Sripuvirus,* and *Sunhavirus*, *n* = 3), *Phenuiviridae* (genus *Uukuvirus*, *n* = 1), *Togaviridae* (genus *Alphavirus*, *n* = 1), *Flaviviridae* (genus *Orthoflavivirus*, *n* = 1), and *Nairoviridae* (genus *Orthovairovirus*, *n* = 1) (Fig. 2; Table S2).

We found six discrepancies between previously available serological classifications and our genomic data (Table S1). Kununurra virus, previously classified in the *Rhabdoviridae* family, was reassigned to the genus *Orthobunyavirus*, *Peribunyaviridae* family. Matariya virus, previously classified as *Orthobunyavirus-like,* was genetically classified as the genus *Sunrhavirus*, *Rhabdoviridae* family. Lake Clarendon virus, formerly assigned to the *Reoviridae* family, was reclassified as a member of the genus *Orthonairovirus*, *Nairoviridae* family. Ndelle virus, previously categorized within the *Orbivirus* genus, was reassigned to the genus *Orthoreovirus, Spinareoviridae* family. Arboledas virus, previously classified into the *Phlebovirus* genus, was reassigned to the genus *Pacuvirus, Peribunyaviridae* family. Lastly, Lanjan virus, originally classified as *Orthobunyavirus-*like*,* was reassigned to the genus *Uukuvirus*, *Phenuiviridae* family.

### Genomic characterization of orbivirus and orthoreovirus isolates

We characterized the genomes of orbivirus and orthoreovirus isolates that we sequenced. Orbiviruses (*Orbivirus* genus, *Sedoreoviridae* family) exhibited a typical genomic organization of 8–10 linear, double-stranded RNA (dsRNA) segments (Fig. 2). Complete ORF sequence lengths ranged from 16,423 to 18,318 nucleotides, with individual segments varying from 585 to 3,978 nucleotides (Fig. 2; Table S4). Each segment was predicted to encode a single protein, including structural proteins (VP1–VP7) and four non-structural proteins (NS1–NS4). In some orbiviruses (i.e., Acado, Bauline, Cape Wrath, Gomoka, Huacho, Jacareacanga, Mono Lake, Monte Dourado, Mykines, Nugget, Sixgun City, Tindholmur, and Yaquina Head), segment 9 uniquely encodes VP6 and NS4 proteins in two distinct ORF regions (Fig. 2; Table S4). We also sequenced the Ndelle virus, a member of the *Orthoreovirus* genus, *Spinareoviridae* family. Like the orbiviruses, the Ndelle virus possessed 10 linear dsRNA segments, but with a larger coding genome length of 22,843 nucleotides. The individual segments ranged from 3,870 to 1,098 nucleotides and were predicted to encode 10 proteins (Fig. 2; Table S4).

### Genomic characterization of bunyavirus isolates

We sequenced and characterized the genomes of 12 peribunyavirids (genera *Orthobunyavirus* and *Pacuvirus, Peribunyaviridae* family), which exhibited typical three-segmented, negative-sense RNA organization (Fig. 2). Their genomes comprised the large (L) segment encoding L protein, the medium (M) segment encoding glycoproteins (GPC, including Gn and Gc), and the small (S) segment encoding a nucleoprotein (N). The segment S of Bobia, Botambi, Brus Laguna, Inini, Kununurra, Olifantsvlei, Ossa, and Xingu viruses also encoded a non-structural protein (NSs) ORF ranging from 204 to 393 nucleotides. Genome lengths ranged from 6,758 to 7,170 nucleotides for the L segment, 4,353 to 5,000 nucleotides for the M segment, and 836 to 1,184 nucleotides for the S segment, with genome lengths between 11,947 and 13,119 nucleotides (Table S4).

We also sequenced the Lanjan virus, which possessed a tri-segmented, negative-sense RNA genome. However, Lanjan virus differs from peribunyavirids primarily in its S segment, exhibiting a unique ambisense strategy that encodes both N and NSs, a characteristic common to members of the *Phenuiviridae* family. The Lanjan virus genome comprised 6,526 nucleotides for the L segment, 3,269 nucleotides for the M segment, and 1,725 nucleotides for the S segment, with a total genome of 11,520 nucleotides. The last tri-segmented, negative-sense RNA virus sequenced in our study was Lake Clarendon virus from the genus *Orthonairovirus* (*Nairoviridae* family) that exhibited a genome of 18,499 nucleotides split into 12,135 nucleotides for the L segment, 4,578 nucleotides for the M segment, and 1,786 nucleotides for the S segment.

### Genomic characterization of rhabdovirus, flavivirus, and alphavirus isolates

We characterized the genomes of three rhabdoviruses (*Rhabdoviridae* family), which possessed typical negative-sense, single-stranded RNA genomes ranging from 10,881 to 12,044 nucleotides. These genomes encoded five structural proteins (N, P, M, G, and L) and additional proteins derived from alternative ORFs (Fig. 2; Table S4). We also characterized Cowbone Ridge virus (*Orthoflavivirus* genus, *Flaviviridae* family), which had a positive-sense non-segmented RNA genome of 10,217 nucleotides. This genome encoded a unique polyprotein of 10,176 nucleotides that produces both structural and non-structural proteins. Finally, we characterized Zingilamo virus (*Alphavirus* genus, *Togaviridae* family), which had a single-stranded, positive-sense RNA genome of 11,193 nucleotides, encoding two non-structural polyproteins (nsP1 and nsP2), and one structural polyprotein (Fig. 2; Table S4).

### Evolutionary history of reoviruses

**Fig 3:**
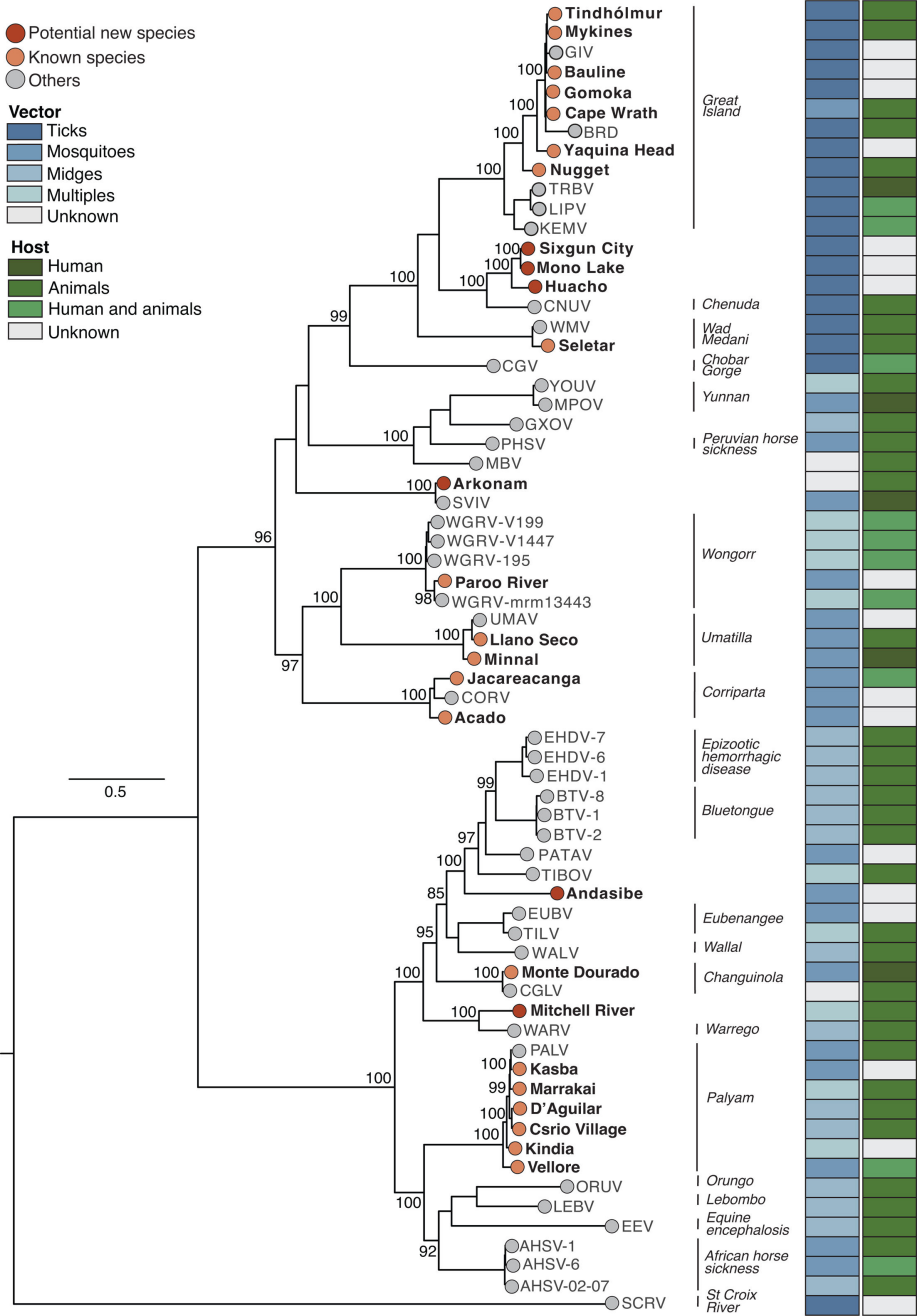
Maximum-likelihood phylogenetic tree of the genus *Orbivirus* (*Sedoreoviridae* family)*.* This phylogenetic tree was inferred using amino acid sequences of the T2 subcore shell protein (VP2) and the third-largest viral protein (VP3). The analysis included 26 newly characterized genomes and 41 representative orbiviruses publicly available. Tip colors indicate the classification of the newly characterized viruses, with red representing potential novel species and orange denoting known species. Gray tips correspond to other *Orbivirus* species. Vertical lines indicate classified *Orbivirus* species. Colored columns indicate the primary host taxon (e.g., humans, animals, or both), and major vector groups (ticks, mosquitoes, midges, or multiple vectors). The tree was midpoint-rooted for clarity, and bootstrap values (based on 1,000 replicates) are shown on principal nodes. Scale bar indicates the evolutionary distance of substitutions per amino acid site. The GenBank accession numbers and full names of all sequences used in this figure are described in Table S5.

We examined the evolutionary history of orbiviruses and the orthoreovirus characterized in this study. The newly sequenced orbiviruses were distributed across multiple clades within the *Orbivirus* genus (Fig. 3). Cape Wrath, Mykines, Nugget, Bauline, Yaquina Head, Tindholmur, and Gomoka clustered with members of the *Orbivirus magninsulae* species (formerly Great Island virus). These viruses are primarily associated with tick vectors (i.e., *Argas*, *Ornithodoros*, and *Ixodes* species), infect seabirds and rodents, and are linked with neurological disease in humans (37, 38). Their distribution spans the North Atlantic region, including Iceland, the Faroe Islands, the British Isles, and Scandinavia (39, 40) (Fig. 3). D’Aguilar, Kasba, Marrakai, Kindia, and Vellore grouped with viruses from *Orbivirus palyamense* (formerly Palyam virus). These viruses are transmitted by biting midges (*Culicoides*) and Culicine mosquitoes, primarily infecting cattle and sheep in Africa, Asia, and Australia (41–43). Some viruses in the *Orbivirus palyamense* species are associated with abortion and teratogenesis in cattle and other ruminants (43–45), but there is no evidence of human infection.

Based on ICTV taxonomic criteria (e.g., <78% amino acid identity in RdRp or <83% amino acid identity in VP3) for orbivirus species, we determined that seven viruses may represent novel *Orbivirus* species: Andasibe, Arkonam, Mono Lake, Sixgun City, Huacho, Yaquina Head, and Nugget (Fig. 3; Fig. S1). Arkonam virus, previously classified as *Orbivirus trinidadense* (formerly *Ieri virus*) grouped with Sathuvachari virus (an unclassified orbivirus) with amino acid divergence of 99.4% and 99.8% in VP1 and VP3, respectively. This high similarity indicates that Arkonam and Sathuvachari viruses belong to the same *Orbivirus* species, although both remain unclassified. Both viruses share a common ancestor with other species-level viruses, including *Peruvian horse sickness virus* (*Orbivirus gammaequi*), *Yunnan virus* (*Orbivirus yunnanense*), *Chobar Gorge virus* (*Orbivirus chobarense*), *Wad Medani virus* (*Orbivirus wadmedaniense*), *Chenuda virus* (*Orbivirus chenudaense*), and *Great Island virus* (*Orbivirus magninsulae*) (Fig. 3). Similarly, Sixgun City, Mono Lake, and Huacho viruses, previously classified as *Orbivirus chenudaense* (formerly *Chenuda virus*), exhibited amino acid identities ranging from 72.1% to 72.8% in VP1 and 62.6% to 64.4% in VP3, also suggesting new orbivirus species.

Next, we identified Ndelle reovirus as a member of the *Mammalian orthoreovirus* (MRV) species, based on the ICTV species demarcation criteria (32), as it shares 98.8%–98.9% amino acid identity with other MRV strains and clusters with MRV types 1–3. MRVs are known for their broad host range, which includes humans, rodents, bats, livestock, and other wild mammals nearly worldwide (46) (Fig. S2). MRV human infections are often asymptomatic or cause mild respiratory and gastrointestinal disease, although severe cases like encephalitis and acute respiratory distress syndrome can occur, particularly in immunocompromised individuals (47–49). In livestock, MRVs can cause gastroenteritis, neurological disorders, and respiratory diseases, particularly in calves and piglets (50, 51).

### Evolutionary history of bunyaviruses

**Fig 4:**
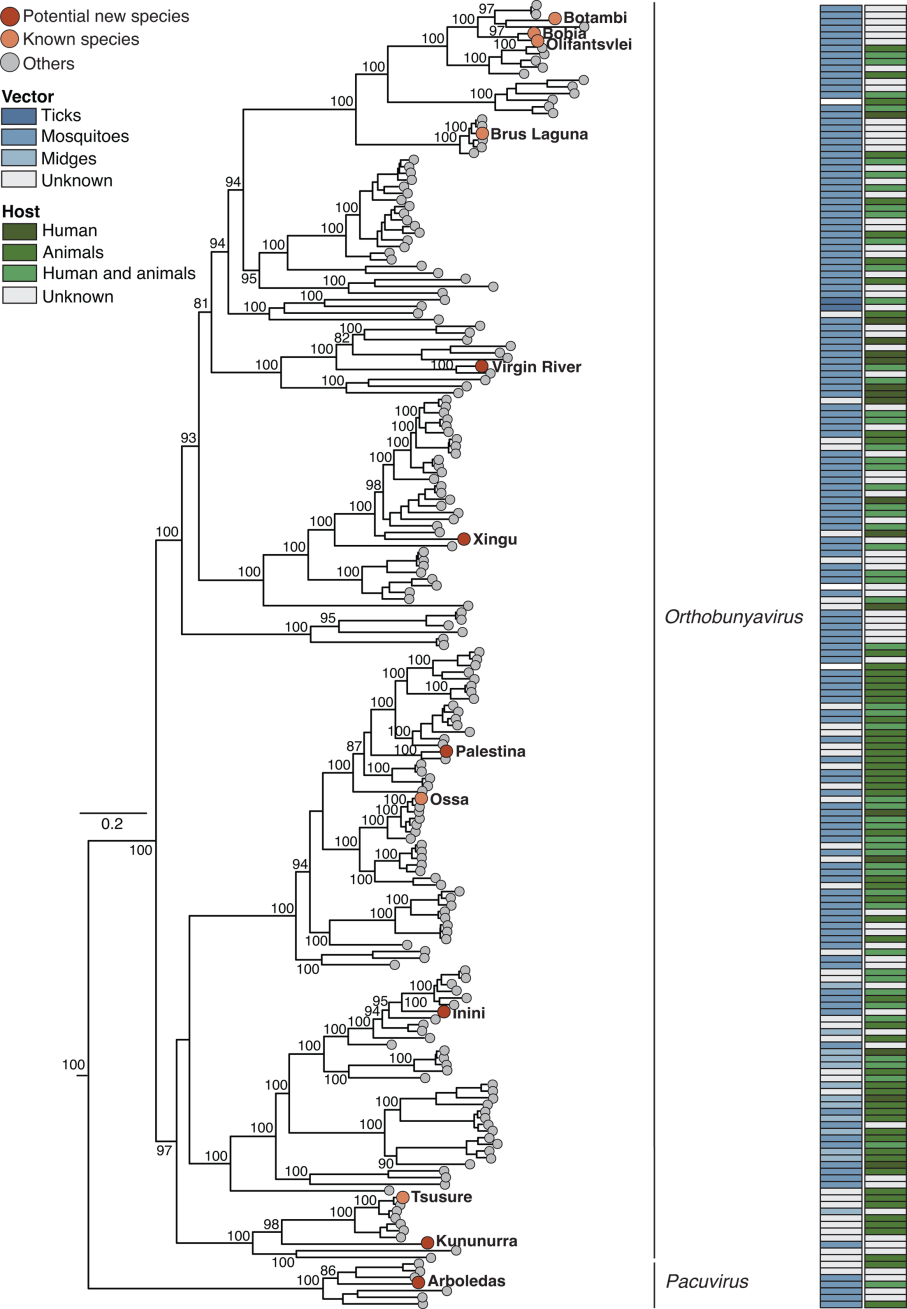
Phylogeny analysis of the *Peribunyavirus* family*,* genera *Orthobunyavirus* and *Pacuvirus.* The maximum likelihood phylogenetic tree was inferred using amino acid sequences of the L protein. The analysis included newly characterized sequences and *Peribunyavirus* sequences retrieved from ICTV resources (*n* = 205) (57). Tip colors indicate the classification of the newly characterized viruses, with red representing potential novel species and orange denoting known species. Gray tips correspond to other *Orthobunyavirus and Pacuvirus* species. The tree was midpoint-rooted for clarity, and bootstrap values (based on 1,000 replicates) are presented on major nodes. The scale bar represents the number of amino acid substitutions per site. The GenBank accession numbers and full names of all sequences used in this figure are listed in Table S5. Diagram colors indicate the primary host taxon of each virus (e.g., humans, animals, or both), while major vector groups (ticks, mosquitoes, midges, or multiple vectors) are also annotated.

The 12 newly characterized peribunyavirids were distributed across the *Orthobunyavirus* genus (*n* = 11) and the *Pacuvirus* genus (*n* = 1) (Fig. 4). Based on the species demarcation criteria for orthobunyaviruses (less than 96% identity in the complete amino acid sequence of the L protein), we propose five new species: Virgin River, Palestina, Inini, Kununurra, and Xingu viruses (Fig. 4; Fig. S1). Virgin River, Palestina, Inini, and Xingu viruses were previously assigned to *Orthobunyavirus tacaiumaense, Orthobunyavirus minatitlanense*, and *Orthobunyavirus manzanillaense*, and *Orthobunyavirus bunyamweraense,* respectively, based on serological data (Table S1) (52–55). Moreover, Kununurra virus was initially classified within the *Rhabdoviridae* family (56). However, all of these viruses meet the current criteria for reclassification as distinct orthobunyavirus species (Fig. S1) (57, 58). These viruses have diverse vector and host distributions: Inini virus was isolated from a black-necked aracari (*Pteroglossus aracari*) in French Guiana in 1973, Virgin River virus was detected in *Anopheles freeborni* captured in Arizona in 1974, and Palestina virus was isolated from *Culex paracrybda* collected in Ecuador in 1975. Xingu virus was isolated from fatal human cases with hepatitis manifestations in Mato Grosso State, Brazil. At present, the vector of the Xingu virus remains unknown, and it has been serologically grouped within the Bunyamwera serogroup (59). Additionally, Kununurra virus was identified in a pool of *Aedeomyia catasticta* mosquitoes collected in Australia in 1973 (56). We confirmed that Botambi, Tsuruse, Brus Laguna, Bobia, Ossa, and Olifanstvlei viruses are previously described orthobunyavirus species (53, 55, 57, 58). These viruses have been isolated from samples of mosquitoes, birds, and humans from Africa, Asia, and Central America (Table S5). Additionally, we determined that Arboledas virus clusters with basal sequences within the clade that includes Caimito virus, Tapirapé virus, and Santarem virus in the *Pacuvirus* genus (60). These viruses have been detected in *Lutzomyia* sandflies and *Oryzomys* rodents, as well as in marsupials of the *Didelphis* genus (60). We propose Arboledas virus as a novel species based on ICTV criteria, which define species-level demarcation as pairwise evolutionary distances >0.1 using the WAG model on concatenated amino acid sequences of all three segments (32). Arboledas virus shows a minimum distance of 0.398 from its closest relative (Tapirape virus) and 0.410 from *Pacuvirus caimitoense*, the representative species of this genus (Fig. 4; Fig. S1).

In addition, based on our reassortant analysis, we identified two reassortant viruses within the *Peribunyaviridae* family, *Orthobunyavirus* genus: Ossa virus, which possesses an M segment derived from the Madrid virus, and Brus Laguna virus, which exhibits unique S and L segments and an M segment derived from the Alajuela virus (Table S6).

We determined that Lake Clarendon virus clusters and shares 99.6% amino acid identity with Vinegar Hill virus (*Orthonairovirus australiaense*) within the genus *Orthonairovirus*, family *Nairoviridae* (Fig. S3 and S4). Based on the species demarcation criterion of <93% amino acid identity in the L segment, Lake Clarendon virus is not considered a new *orthonairovirus* species (32). Both viruses are associated with *Argas robertsi* ticks collected in Australia. Vinegar Hill virus was isolated from a pool of five female *Argas robertsi* collected from a cattle egret (*Bulbulcus ibis*) rookery (61). Antibodies specific to Vinegar Hill virus were detected in 3.2% (13 of 401) of seabird sera and 1% (1 of 101) of human sera in Australia (62). Experimental infection has shown that Vinegar Hill virus can cause infection and death in cattle egrets (62). In addition, Lanjan virus grouped and shared 90% amino acid identity with Tongren Perib Tick Virus 2, an unclassified *Peribunyaviridae* (63), in the *Uukuvirus* genus, *Phenuiviridae* family. Lanjan virus was isolated from a pool of *Dermacentor auratus* ticks in Malaysia in 1960 (64). Based on the current ICTV species demarcation criteria for phenuiviruses (65), we propose Lanjan virus as a new uukuvirus species, supported by its amino acid identity <95% in the L protein with Tongren Perib tick virus 2 (Fig. S3).

### Evolutionary history of rhabdoviruses, flavivirus, and alphavirus

**Fig 5:**
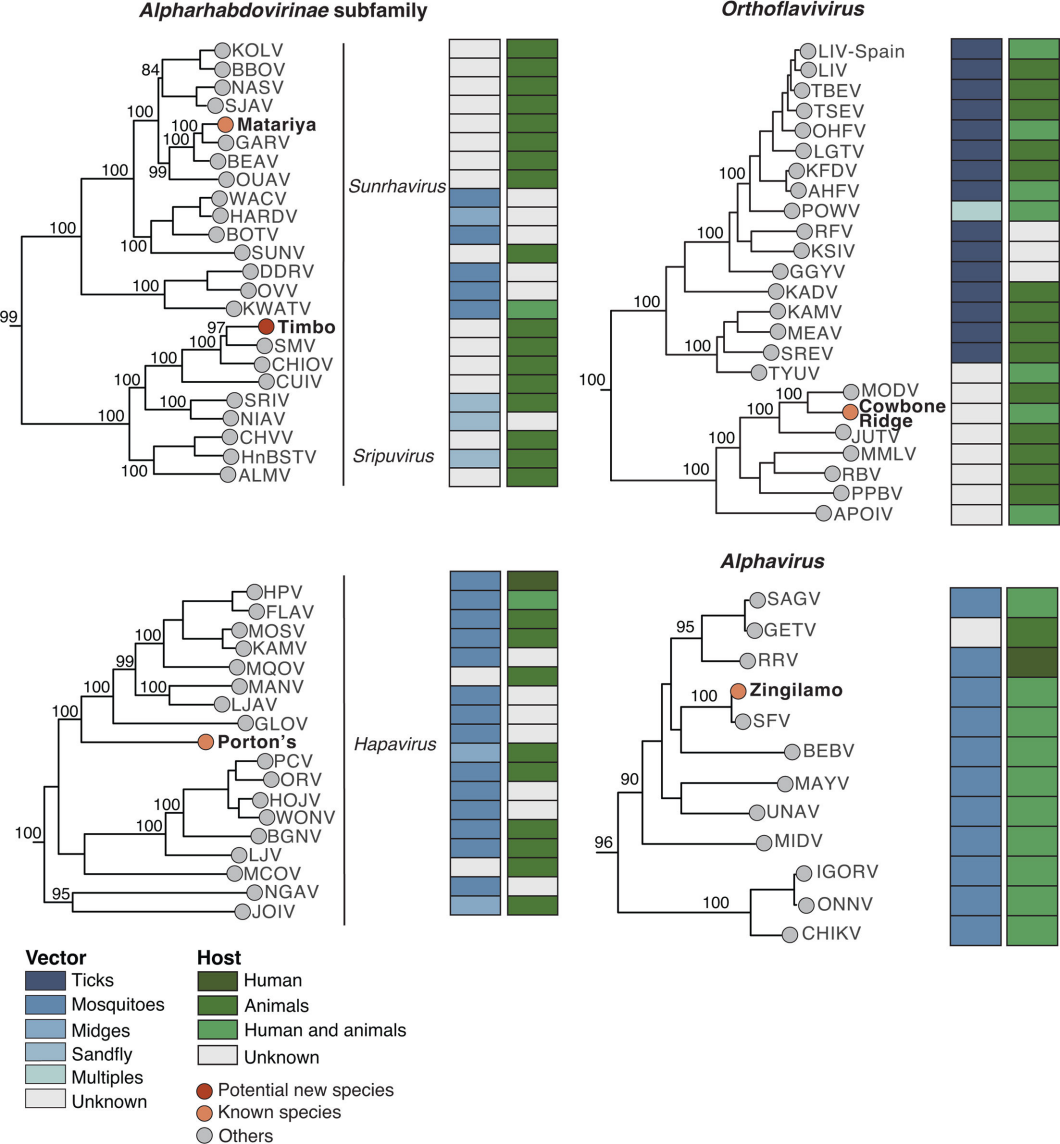
Phylogenetic analysis of *Alpharhabdoviridae* subfamily, genera *Sunhavirus*, *Sripuvirus*, and *Hapavirus*; *Flaviviridae* family, genus *Orthoflavivirus*; and *Togaviridae* family, genus *Alphavirus*. The maximum likelihood phylogenetic tree inferred for the *Alpharhabdoviridae* subfamily was based on amino acid sequences of newly identified *Rhabdoviridae* family viruses, along with 315 complete coding sequences of the L segment (L protein) from other members. *Flaviviridae* family includes amino acid sequences from the RdRp (*NS5* or *NS5B*) of *Flaviviridae* members (*n* = 94), and *Togaviridae* family was inferred from a conserved region of the envelope protein gene nucleotide sequences (*n* = 48). All sequences were retrieved from ICTV resources for each family. Complete trees are provided in Fig. S5 to S7 of the supplementary material. Tip colors indicate the classification of the newly characterized viruses, with red representing potential novel species and orange denoting known species. Gray tips correspond to other species. The trees were midpoint-rooted for clarity, with bootstrap values (based on 1,000 replicates) displayed on major nodes. The scale bar represents the number of amino acid substitutions per site. GenBank accession numbers and full sequence names are listed in Table S5. Color coding in the diagram indicates the primary host taxon of each virus (e.g., humans, animals, or both), with annotations for major vector groups (e.g., ticks, mosquitoes, midges, or multiple vectors).

We examined the evolutionary relationships of three newly sequenced rhabdoviruses and found that they belong to the genera *Hapavirus* (Porton’s virus), *Sunhavirus* (Matariya virus), and *Sripuvirus* (Timbo virus) within the *Alpharhabdovirus* subfamily (Fig. 5A; Fig. S5). *Sunhavirus* species have been isolated from mosquitoes (*Culicinae*), midges, and birds in Africa, North America, and Oceania (66, 67). *Sripuvirus* species have been isolated from lizards and phlebotomine sandflies in Africa, Asia, South America, and Australia (68–70). *Hapavirus* species have been isolated from passerine birds, *culicine* mosquitoes, and midges (*Culicoides* spp.) in America, Africa, Asia, and Oceania (68, 71–73). Based on the *Rhabdoviridae* species demarcation genome criteria (70), we propose Timbo virus as a new species within the *Sripuvirus* genus, as it shares 77.3% amino acid identity in the N protein, 72.8% in the L protein, and 71.9% in the G protein with Sena Madureira virus (Fig. S1). Additionally, Porton’s virus and Matariya virus were confirmed as established rhabdovirus species, classified as *Hapavirus porton* and *Sunhavirus matariya*, respectively.

We found that Cowbone Ridge virus, already described as *Orthoflavivirus cowboneense*, clusters with and shares 74.8% amino acid identity with *Modoc virus*, a member of the species *Orthoflavivirus commonsense* (Fig. 5B; Fig. S6). Cowbone Ridge virus and related flaviviruses, including Modoc, Jutiapa, Sal Vieja, and San Perlita viruses, have been detected in bats and rodents and are considered flaviviruses with no known vectors (74–77). Lastly, the alphavirus, Zingilamo virus, clusters closely with Semliki Forest virus (SFV), which is known for its broad host range, having been isolated from *Culex* and *Aedes* mosquitoes, chimpanzees, and wild birds in Africa and Asia. SFV has also been linked to fatal human encephalitis following a laboratory-acquired infection in Germany (78). Zingilamo virus shares 99.2% sequence identity with SFV, supporting its classification within the same species, *Alphavirus semliki* (32) (Fig. 5C).

### Potential risk of emergence and human infection

**Fig 6:**
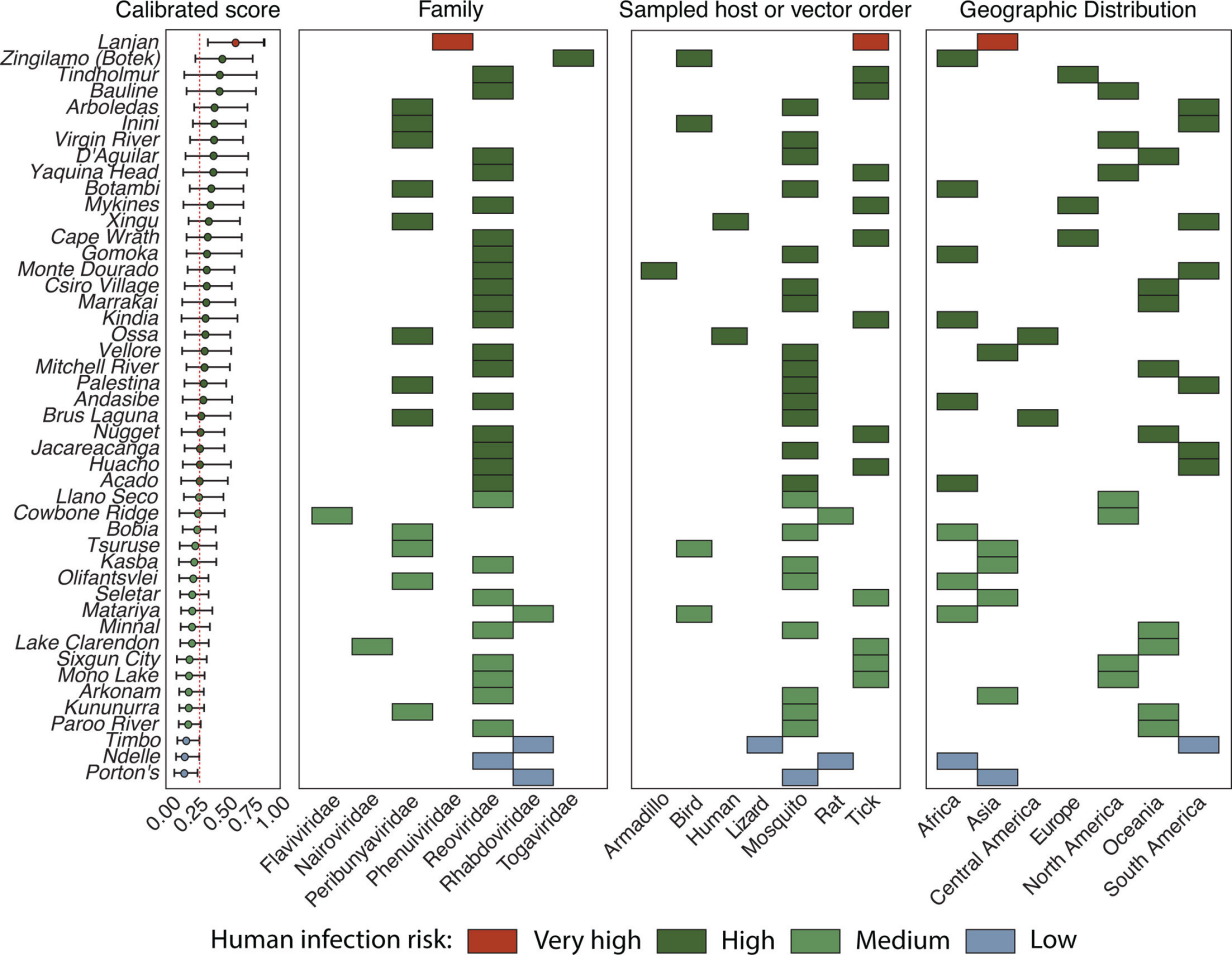
Predicted probability of human infection for newly genomic characterized arboviruses based on a genome composition-based model. Points represent the mean calibrated score, with lines indicating 95% confidence intervals. The dashed line marks the threshold score of 0.293, distinguishing high and very high zoonotic potential categories (37). The color scale corresponds to assigned zoonotic potential: low (dark green), medium (light green), high (light blue), and very high (dark blue). The data is distributed across viral families, sampled host or vector orders, and geographic regions. Additional information is provided in Table S8.

We assessed the emergence risk of potentially human-infecting viruses among the arboviruses by using a machine learning model, as previously described (36). Our analysis indicated that the Lanjan virus has the highest (very high) potential for human infection among all the viruses characterized. Additionally, 56.5% (26 of 46) of the arboviruses were classified as having a high potential for human infection, with the highest scores observed for Zingilamo (score = 0.49), Tindholmur, and Bauline viruses (score = 0.47 each). Furthermore, 36.9% (17 of 46) were classified as having medium potential. Lastly, Porton’s and Ndelle viruses (scores = 0.16 and 0.13, respectively) were classified as having a low potential for human infection (Fig. 6). Among the viruses classified as having very high or high potential, 80.8% (21 of 26) were sampled from arthropod vectors, including mosquitoes and ticks. These viruses exhibit a wide geographic distribution and a broad range of host and vector species, and they have been isolated from all continents.

## DISCUSSION

This study provides a genomic and evolutionary characterization of 46 viruses originally classified as arboviruses. Our data contribute to the classification of these viruses into 11 genera and 8 viral families, and we identified 15 potential new viral species. Notably, our data increase the number of available complete ORFs from genomes of orbiviruses, from both known and previously uncharacterized species, by over 50%. Our genomic data confirm most previous serological classifications. However, we observed discrepancies for six viruses, which were subsequently reassigned to different genera and families. Such inconsistencies between genomic and serological methods for viral taxonomy have been reported previously (79–83). Serological approaches rely on antigenic methods (e.g., complement fixation, hemagglutination inhibition, and neutralization assays) and can be affected by several factors, such as the specific antigens used, potential co-infection during viral isolation, and contamination of the viral isolates used in these tests. Furthermore, genomic reassortment among multisegmented viruses (e.g., orthobunyaviruses and orbiviruses) can also impact serological test results. Interestingly, we found that the Ndelle virus, originally classified as an orbivirus and presumably arbovirus, belongs to the *Mammalian orthoreovirus* (*Orthoreovirus* genus, *Spinareoviridae* family). The members of *Mammalian orthoreovirus* are widely distributed and cause mild gastroenteritis and respiratory disease in humans, wild and domestic animals, and are transmitted via the fecal-oral route or through respiratory droplets (47–51). Therefore, the Ndelle virus seems unlikely to be an arbovirus. Collectively, our data demonstrate that viral genome and metagenomic sequence data under strict quality control substantially contribute to our understanding of viral evolutionary history and taxonomy. This genomic information can complement biological properties, such as pathogenicity, host range, and epidemiology (84).

Our findings indicate the potential for some of the arboviruses characterized in this study to cause human infection based on a model using viral genome composition. This predictive model highlights the potential and ability to infect humans that may be used to prioritize biological investigation (36). For instance, we found the highest predictive values for Lanjan, Zingilamo, Tindholmur, and Bauline viruses. Currently, each of these viruses has only one isolate that has demonstrated the ability to replicate in both vertebrate and invertebrate cells, but they have not yet been linked to infections in humans or domestic animals. Therefore, further studies using murine models may contribute to understanding the potential for these viruses to cause disease in mammals. We emphasize this particularly for Tindholmur and Bauline viruses, which are related to the Great Island virus serogroup, including the Tribeč and Kemerovo viruses, which are known to cause central nervous system infections in humans and mouse models (85, 86). However, we emphasize that emergence potential is also strongly linked to ecological opportunities, which are often unpredictable and can be influenced by various bottleneck effects (87–89). For instance, the Zika virus remained obscure for decades after its discovery in 1947, with few human cases. However, its emergence in 2007, attributed to reversions of deleterious founder effect mutations (90), led to major outbreaks between 2014 and 2015 in the Federated States of Micronesia, French Polynesia, and America, including severe outcomes, such as congenital disorders (e.g., microcephaly) and Guillain–Barré syndrome (91, 92).

Our study has several limitations. First, we did not conduct experimental animal studies to validate the pathogenic potential of the characterized arboviruses. Although some of these viruses have been studied in murine models, most require further *in vivo* characterization. Second, the nearly complete ORFs generated provide important information regarding taxonomy, evolutionary history, and, to some extent, phenotype and host range. Additionally, this information can be useful for designing molecular tests such as reverse transcription-PCR. However, some regions of these viral genomes, particularly untranslated regions, remain to be sequenced and characterized. Third, the machine learning-based predictions of human infection rely exclusively on genomic information. Consequently, this approach has several limitations due to the uncertainty regarding the model’s assumptions, the quality of the training data, and the absence of complex host-pathogen interactions that influence spillover risk (93). Overall, future studies that focus on examining the biological properties of these viruses may be important for anticipating and preparing for the potential emergence of these or related viruses.

In conclusion, our genomic and evolutionary characterization of historical arbovirus isolates improves our understanding of the diversity, evolution, taxonomy, and potential risk of human infection of arboviruses. This information can inform studies focused on biological characteristics and contribute to developing molecular and serological methods for detecting these viruses. Lastly, the knowledge generated in this study can strengthen our preparedness for future arbovirus emergence and reemergence.

## Data Availability

This study did not develop any custom code. All viral genome sequences were submitted to GenBank under accession numbers PV804325–PV804639.

## SUPPLEMENTAL MATERIAL

Supplemental material jvi.01214-25-s0001.pdf Figures S1 to S7; Tables S1 to S8.

ASM does not own the copyrights to Supplemental Material that may be linked to, or accessed through, an article. The authors have granted ASM a non-exclusive, world-wide license to publish the Supplemental Material files. Please contact the corresponding author directly for reuse.
