# Supplementary material for: Genomic characterization of arboviruses discovered between the 1950s and 1980s
Source: J Virol. 2025 Sep 8;99(10):e01214-25. doi: 10.1128/jvi.01214-25 (PMC12548411; doi:10.1128/jvi.01214-25)
Supplement: Supplemental material — Figures S1 to S7; Tables S1 to S8. [file jvi.01214-25-s0001.pdf]

Supplementary appendix

Supplementary Figures

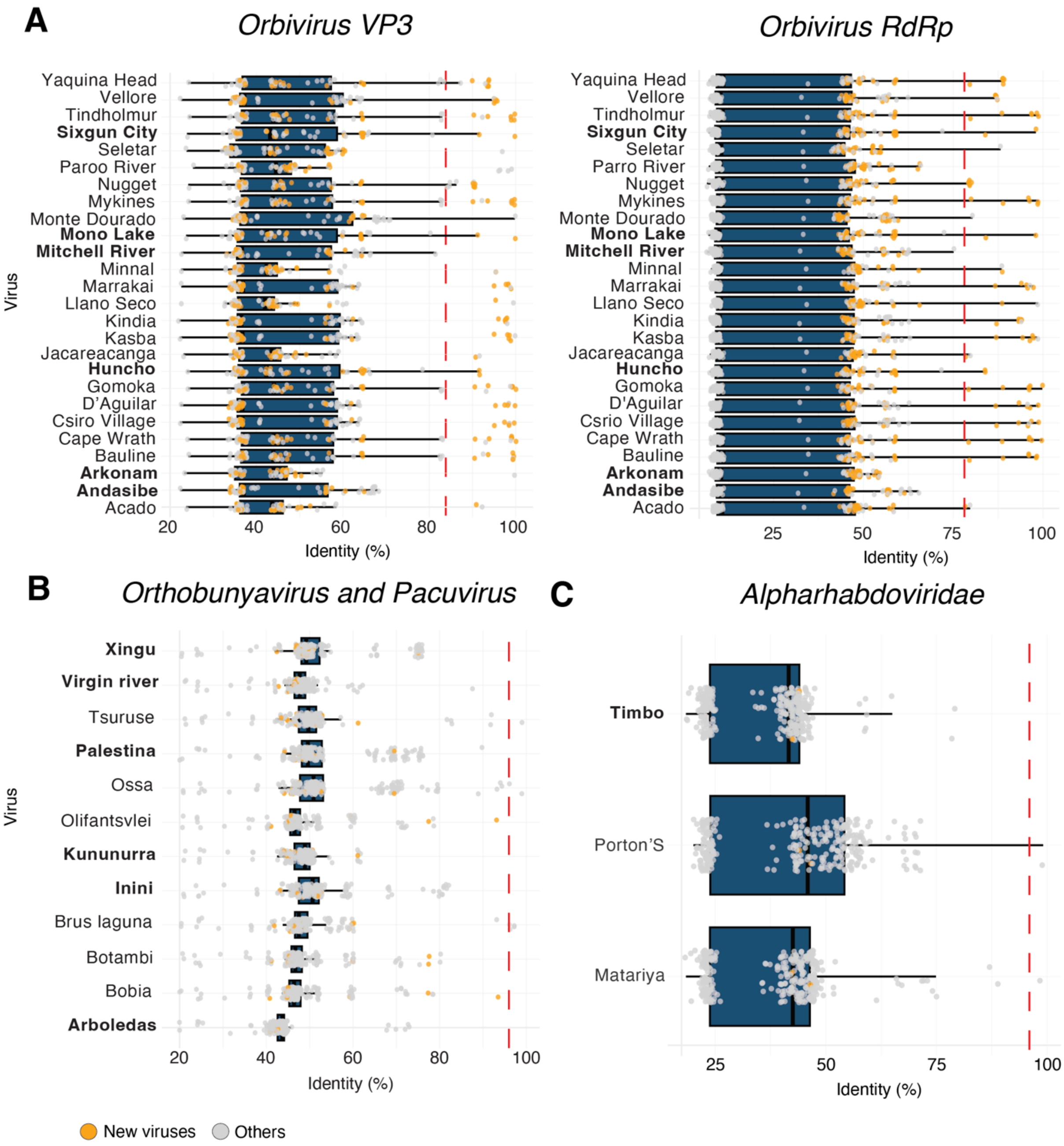

Supplementary Figure 1. Boxplot of pairwise genetic distances among newly sequenced and reference viruses. The boxplot illustrates the distribution of genetic distances for each virus, calculated from pairwise comparisons. The boxes represent the interquartile range (IQR), with the horizontal line indicating the median. Individual data points are plotted as dots, with gray dots representing pairwise distances among general viruses and orange dots highlighting comparisons involving newly sequenced viruses. A dashed red line denotes the species demarcation threshold for each viral family, as defined by the International Committee on Taxonomy of Viruses (ICTV) (1).

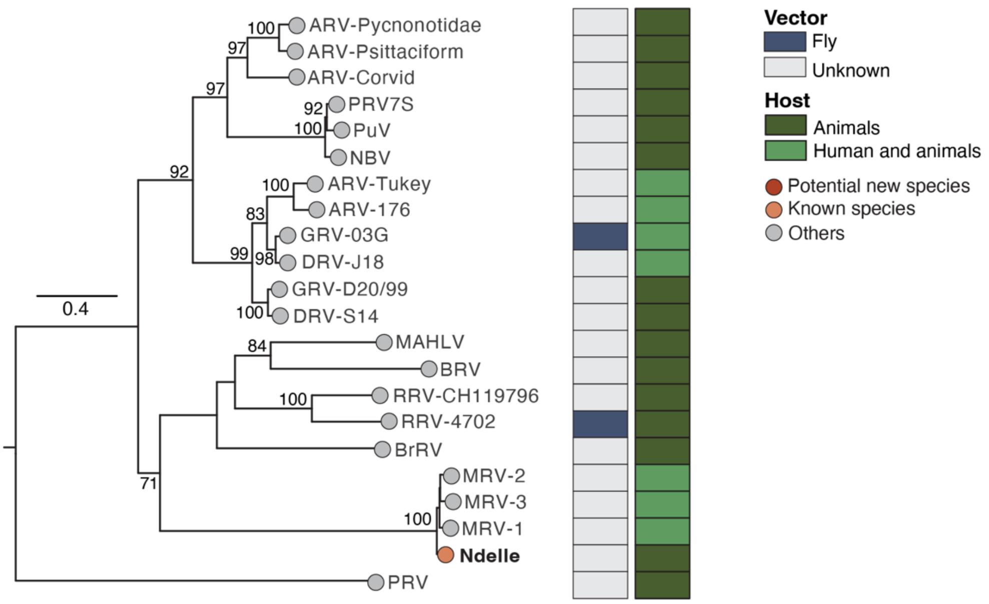

Supplementary Figure 2. Maximum-likelihood phylogenetic tree of genus *Orthoreovirus* (*Sedoreoviridae* family*).* This phylogenetic tree was inferred using amino acid sequences of the outer clamp proteins and the Le and Gascuel amino acid substitution model with invariable sites and a gamma distribution for rate heterogeneity (LG+I+G). The analysis included one newly characterized genome and 21 representative orthoreovirus genomes publicly available from ICTV resources. Ndelle reovirus was classified within the *Mammalian orthoreovirus* (MRV) species based on ICTV species demarcation criteria: for conserved core proteins, species-level identity is defined as >85% amino acid identity within a species and <65% between species; for more variable outer capsid proteins, thresholds are >55% within species and <35% between species (2). Tips are colored to distinguish between potential novel species and known species. The tree was midpoint-rooted for clarity, and bootstrap values (based on 1,000 replicates) are shown on principal nodes. Scale bar indicates the evolutionary distance of substitutions per amino acid site. The GenBank accession numbers and full names of all sequences used in this figure are described in **Table S5**. Colored columns indicate the primary host taxon (e.g., humans, animals, or both), and major vector groups (ticks, mosquitoes, midges, or multiple vectors).

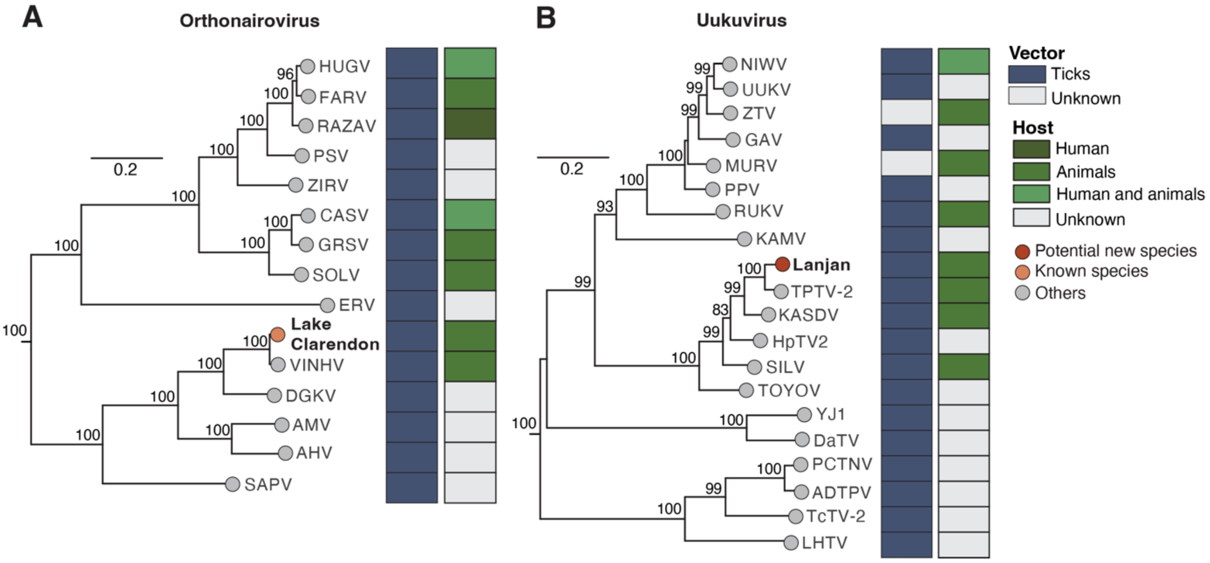

Supplementary Figure 3. Maximum-likelihood phylogenetic tree of genus *Orthonairovirus* (*Nairoviridae* family*)* and *Uukuvirus* (*Phenuiviridae* family). The maximum likelihood phylogenetic tree for the *Nairoviridae* family was inferred using amino acid sequences of newly identified viruses, along with 14 complete coding sequences of the L (RNA-dependent RNA polymerase, RdRp) protein from other family members. The analysis was performed using the Le and Gascuel (LG) amino acid substitution model with invariable sites (I) and a gamma distribution (G) for rate heterogeneity (LG+I+G). The complete phylogenetic tree is provided in **Figure S4**. According to ICTV species demarcation

criteria for this family, viruses sharing <93% amino acid identity in the L protein are considered distinct species. Based on these criteria, Lake Clarendon virus clusters with Vinegar Hill virus and is not considered a novel species (3). For the *Phenuiviridae* family, the analysis included amino acid sequences of the L protein from 19 Uukuvirus genus members, also using the LG+I+G model. All sequences were retrieved from ICTV resources for each viral family. Tree tips are color-coded to distinguish between potential novel species and known species. Tip colors indicate the classification of the newly characterized viruses, with red representing potential novel species and orange denoting known species. Grey tips correspond to other species. Based on ICTV species thresholds for *Uukuvirus* (species demarcation <95% amino acid identity in the L protein), Lanjan virus is proposed as a novel species within this genus (4). The trees were midpoint-rooted for clarity, with bootstrap values (based on 1,000 replicates) displayed on major nodes. The scale bar represents the number of amino acid substitutions per site. GenBank accession numbers and full sequence names are listed in **Table S5**. Color coding in the diagram indicates the primary host taxon of each virus (e.g., humans, animals, or both), with annotations for major vector groups (e.g., ticks, mosquitoes, midges, or multiple vectors).

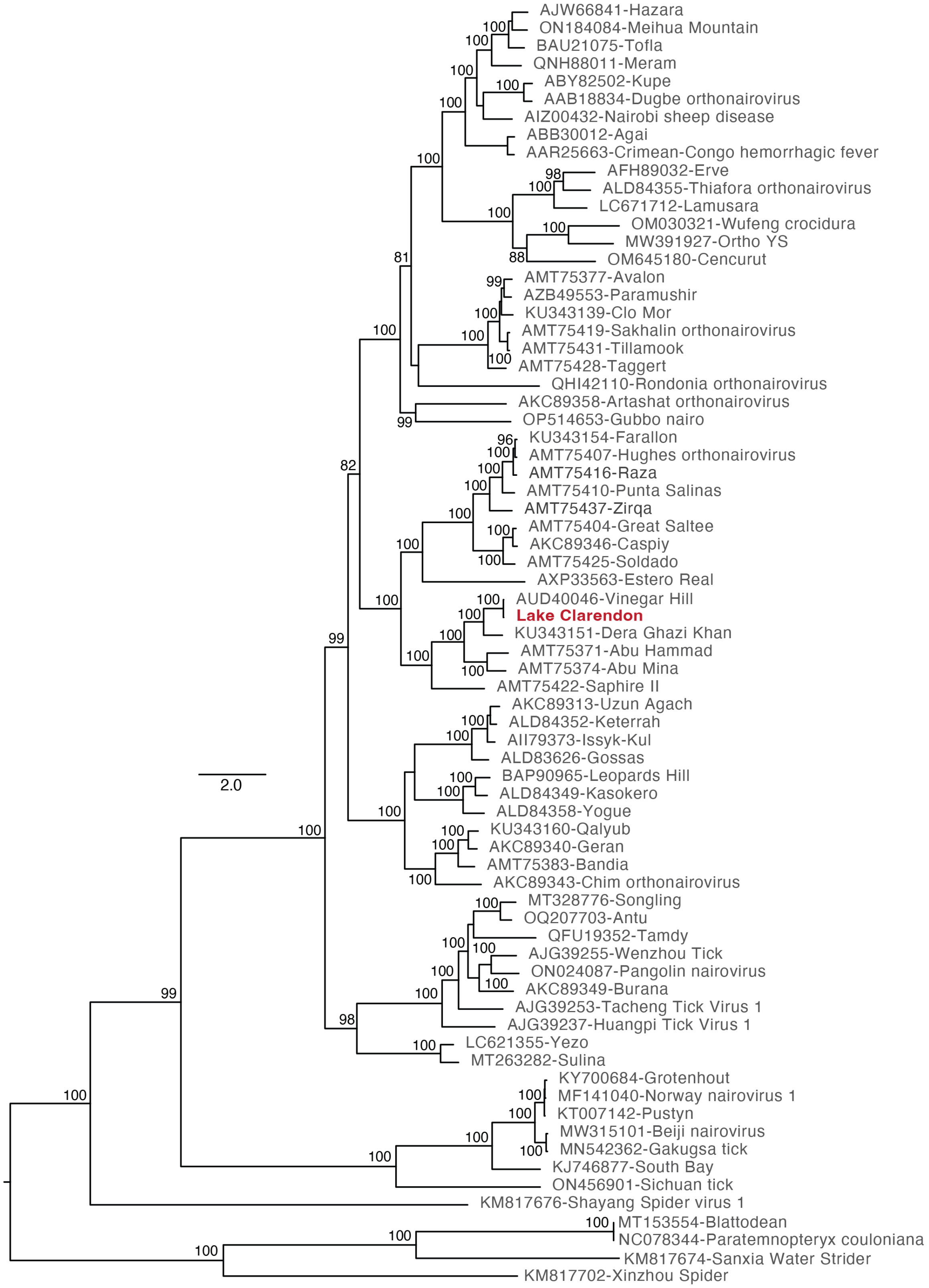

Supplementary Figure 4. Maximum-likelihood phylogenetic tree of genus *Orthonairovirus* (*Nairoviridae* family). This is the complete tree of the *Nairoviridae* family was based on amino acid sequences of newly identified viruses, highlighted in red, along with 71 complete coding sequences of the L (RdRp) protein from other members presented as collapsed in **Figure S3**. Tip labels indicate GenBank accession number and virus name. The tree is midpoint rooted for clarity, with bootstrap support values (1000 replicates) shown for major nodes**.** The scale bar represents the number of amino acid substitutions per site. Bootstrap values based on 1,000 replicates are shown on principal nodes.

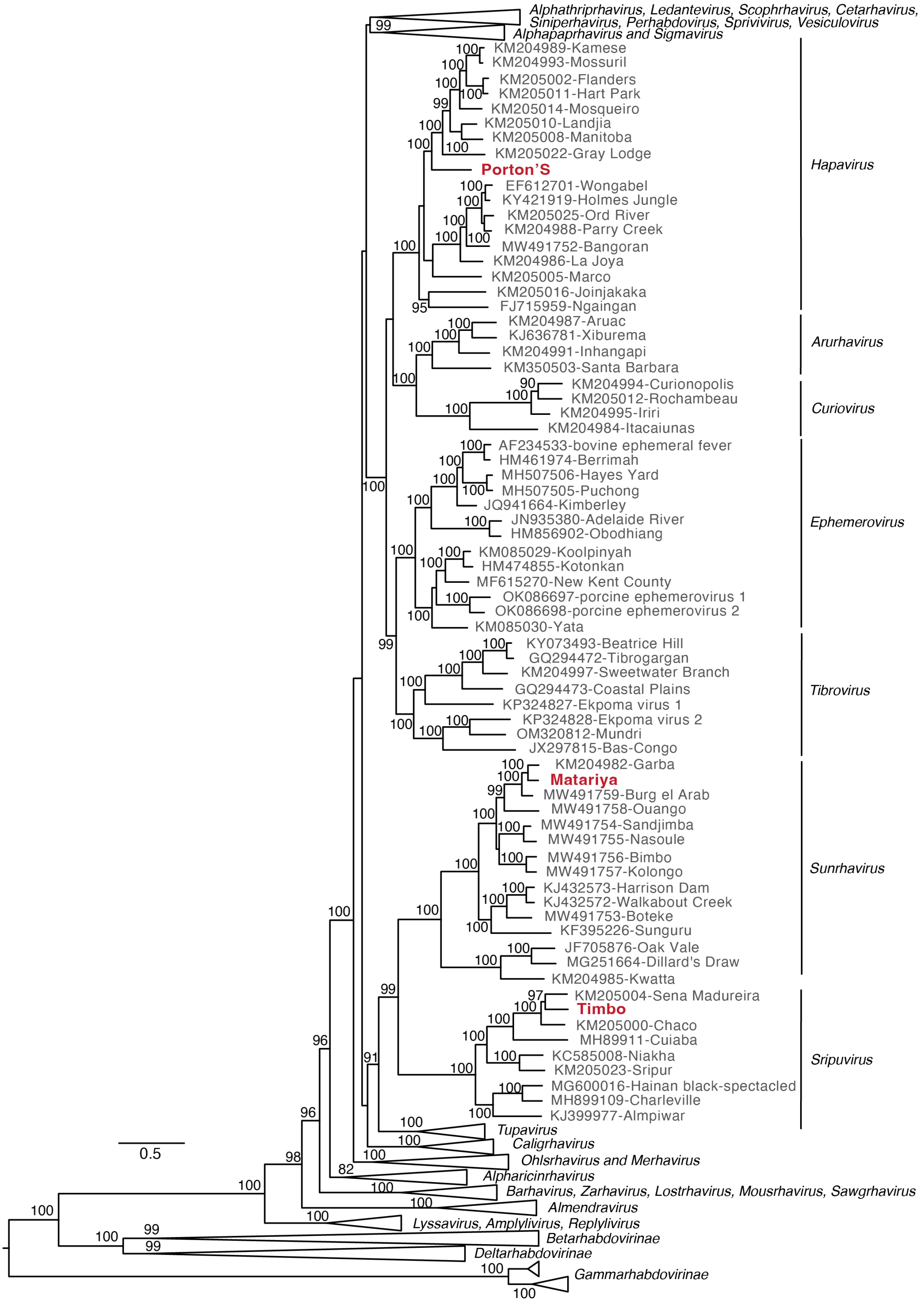

Supplementary Figure 5. Maximum-likelihood phylogenetic tree of *Rhabdoviridae* family. This figure presents the complete maximum-likelihood phylogenetic tree of the *Rhabdoviridae* family, constructed based on amino acid sequences from three newly identified viruses, highlighted in red, along with 312 complete coding sequences (virus names in grey) of the L (RdRp) protein from other family members, with virus names shown in grey. Collapsed clades corresponding to these sequences are presented in **Figure 5**. The analysis was performed using the Le and Gascuel amino acid substitution model with invariable sites and a gamma distribution to account for rate heterogeneity

(LG+I+G). Taxonomic proposals and confirmations: Timbo virus is proposed as a novel species within the *Sripuvirus* genus, based on ICTV species demarcation criteria: ≥5% amino acid divergence in the N protein; ≥10% in the L protein, and ≥15% in the G protein. Porton's virus and Matariya virus are confirmed as previously described species, classified as *Hapavirus porton* and *Sunhavirus matariya*, respectively. *Hapavirus porton* meets thresholds of: ≥5% divergence in N, ≥10% in L, ≥15% in G. *Sunhavirus matariya* meets thresholds of: ≥10% divergence in N, ≥10% in L, ≥15% in G (5).

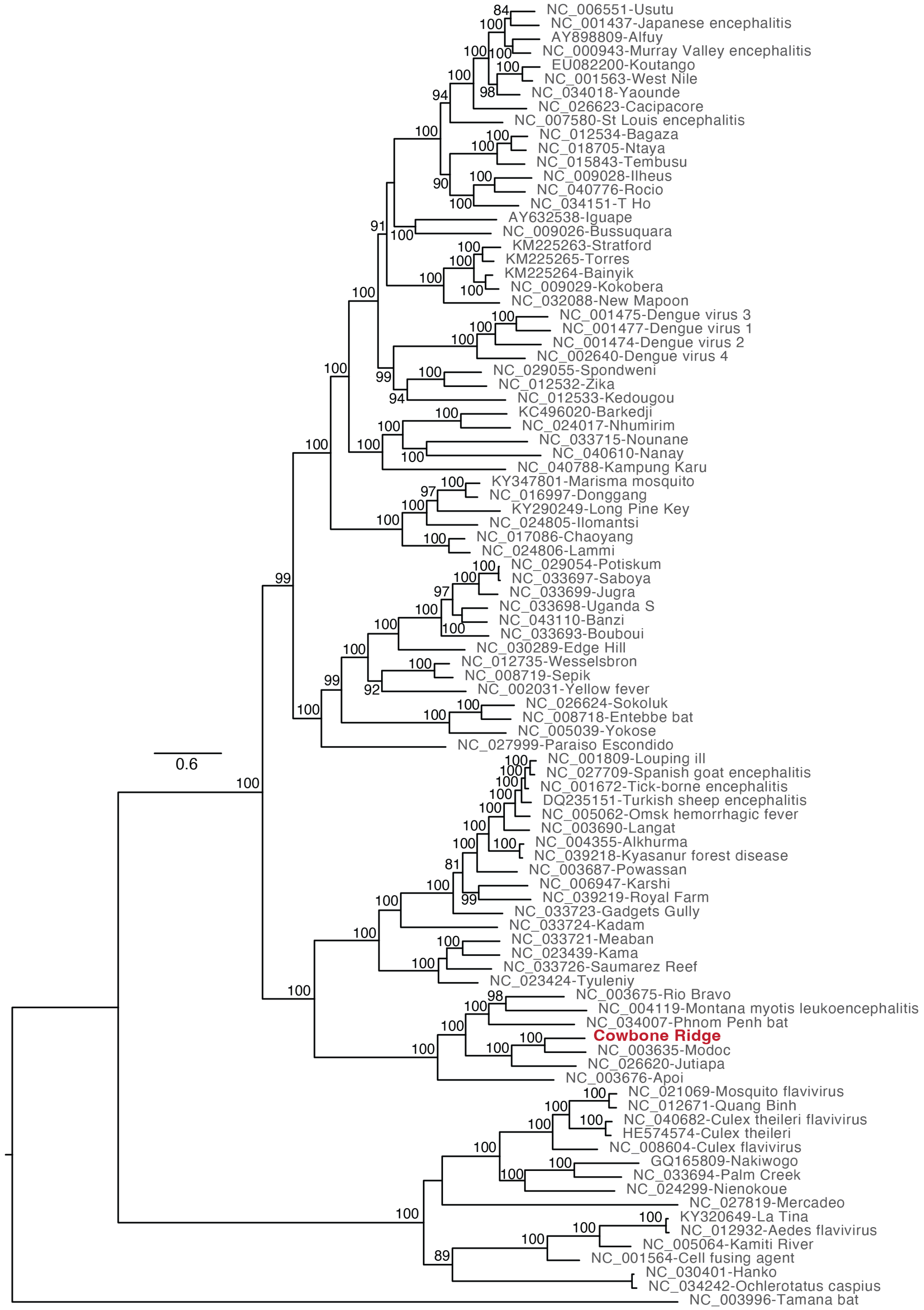

Supplementary Figure 6. Maximum-likelihood phylogenetic tree of genus *Orthoflavivirus* *(Flaviviridae* family). This is the complete phylogenetic tree of the *Flaviviridae* family, inferred from amino acid sequences of newly identified viruses, highlighted in red, along with 93 complete coding sequences of the RdRp (NS5 or NS5B) protein. The analysis was conducted using the Le and Gascuel amino acid substitution model with invariable sites and a gamma distribution for rate heterogeneity (LG+I+G). According to ICTV criteria, *Orthoflavivirus* species are defined based on a combination of genetic, ecological, and antigenic features. Although no strict sequence identity threshold is defined, amino acid divergence in conserved proteins (e.g., NS5) is a key parameter. Cowbone Ridge virus was

previously classified as *Orthoflavivirus cowboneense*, supporting its status as a distinct species (6). The tree is presented in its collapsed form in **Figure 5**. Tip labels indicate GenBank accession number and virus name. The tree is midpoint rooted for clarity, with bootstrap support values (1000 replicates) shown for major nodes. The scale bar represents the number of amino acid substitutions per site. Bootstrap values based on 1,000 replicates are shown on principal nodes.

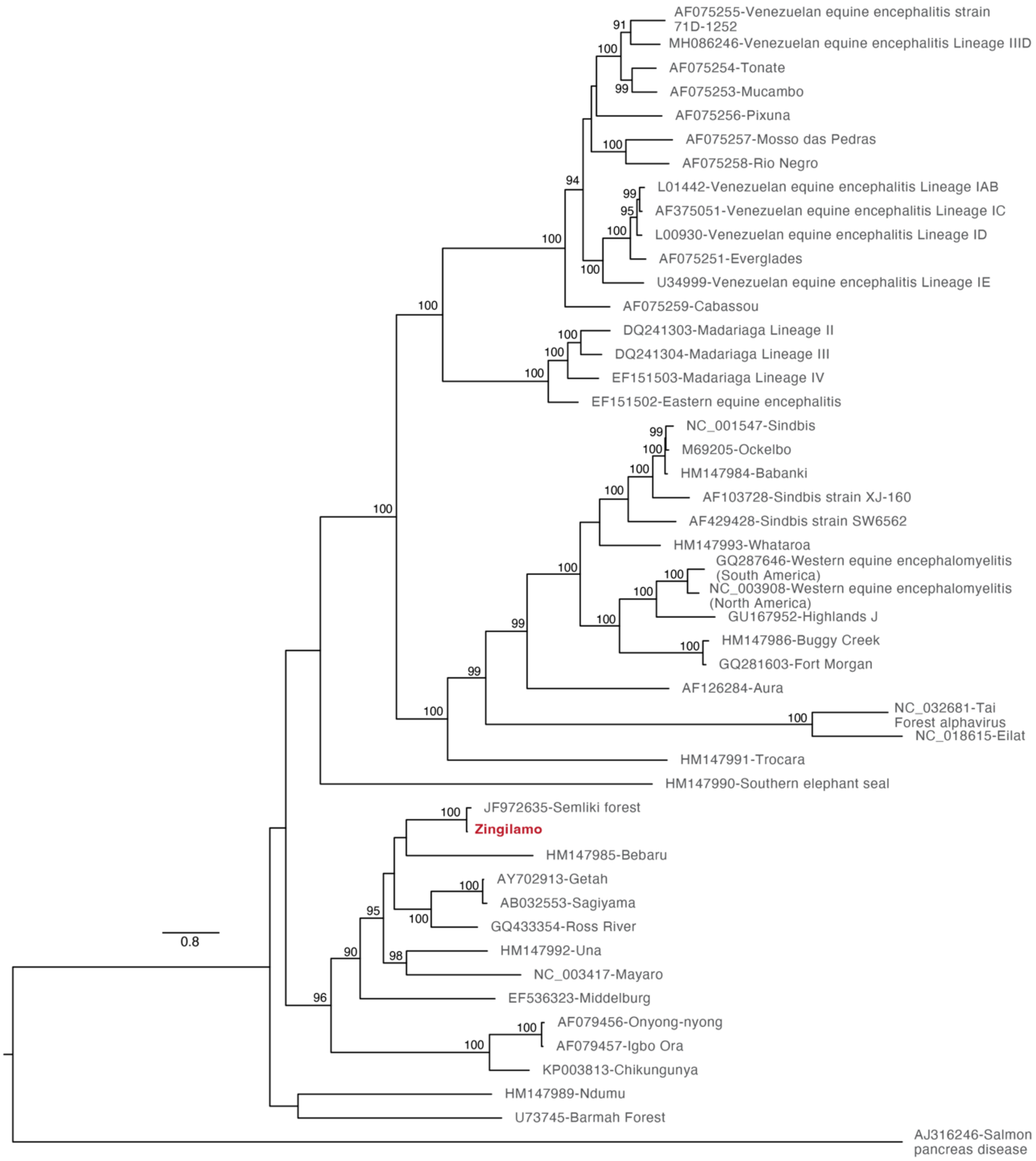

Supplementary Figure 7. Maximum-likelihood phylogenetic tree of genus *Alphavirus* (*Togaviridae* family). This is the complete phylogenetic tree of the *Togaviridae* family, inferred from nucleotide sequences of newly identified viruses, highlighted in red, along with 47 complete coding sequences of the conserved region of envelope protein gene using the General Time Reversible substitution model with invariable sites and a gamma distribution (GTR+I+G) substitution model. The tree is presented in its collapsed form in **Figure 5**. According to ICTV criteria, Alphavirus species are defined based on a combination of genetic, antigenic, ecological, and epidemiological characteristics (7). Although no strict threshold is set, species typically differ by more than 20% in the E1 gene. Zingilamo virus was therefore classified within the same species, *Alphavirus semliki.* Tip labels indicate GenBank accession number and virus name. The tree is midpoint rooted for clarity, with bootstrap support values (1000 replicates) shown for major nodes**.** The scale bar represents the number of nucleotide substitutions per site. Bootstrap values based on 1,000 replicates are shown on principal nodes.

Supplementary Tables

Supplementary Table 1. Serological cross-reactivity data, isolate passage history, and epidemiological metadata for arboviruses analyzed in this study.

Name	Strain Designation	Collection date	Vector/host	Vector common name	Place collected	Antigenic Group	Experimental host and age	Evidence of infection	Passage history	References
Acado	ETH AR 1846-64	22-Oct-1963	*Culex antennatus and Culex univittatus neavi*	Mosquito	Acado, Baro River, Ilubabor Prov., Ethiopia	Corriparta	Mice (nb)	Death	SM 12	(8-10)
Andasibe	DAK AR MG 807	01-Feb-1979	*Anopheles pauliani and Anopheles squamosus*	Mosquito	Andasibe, Madagascar	Ungrouped	Mice (nb)	Paralysis and death	SM 7	(11)
Arboledas	CO AR 170152 (A 40,56)	06-Feb-1984	*Lutzomyia spp.*	Mosquito	Arboledas, Norte de Santander, Colombia	Phlebotomus Fever	Mice (nb and adult)	Illness and Death; Antibody; Viremia and antibody	Vero 2	(12)
Arkonam	IG 10658	30-Jan-1957	*Anopheles subpictus*	Mosquito	Minnal, India	Ungrouped	Mice (nb); embryonated eggs (7 day)	Sickness and death; Death	SM 40	(13)
Bauline	CAN AR 14	23-Jul-1971	*Ixodes uriae (Ixodes putus)*	Tick	Great Island, Newfoundland, Canada	Kemerovo	Mice (nb); chicks (1 day)	Paralysis and death; Paralysis and death	SM 5	(14-16)
Bobia	DAK AR B 1569	14-Feb-1969	*Culex trigripes*	Mosquito	Bobia, Central African Republic	Olifantsvlei	Mice (nb and adult)	Death; Death; Antibody	SM 9	(17)
Botambi	DAK AR B 937	10-Jul-1968	*Culex guiarti*	Mosquito	Botambi village, Ombella-Mpoko, Central African Republic	Olifantsvlei	Mice (nb and adult)	Death; Antibody	SM 3	(18, 19)
Brus Laguna	HBL 7-2CH	1967	*Aedeomyia squamipennis*	Mosquito	Honduras, Central America	-	-	-	SM 7	(20)
Cape Wrath	SCOT AR 20	20-Jun-1973	*Ixodes uriae (Ixodes putus)*	Tick	Clo Mor, Cape Wrath, Scotland	Kemerovo	Mice (nb)	Paralysis and death	SM 3	(16)
Cowbone Ridge	W 10986	10-Jan-1965	*Sigmodon hispidus*	Rat	Cowbone, Hendry County, Florida, USA	Boteke	Mice (nb)	Paralysis and death	SM 5	(21, 22)
Csiro Village	CSIRO 11 (BH 0058)	20-Nov-1974	*Culicoides brevitarsis*	Mosquito	Beatrice Hill, North Territory, Australia	Palyam	Mice (nb)	Paralysis and death	SM 3, Vero 1	(23, 24)
D’Aguilar	B 8112	02-Apr-1968	*Culicoides brevitarsis Kieffer*	Mosquito	Bunya, Queensland, Australia	Palyam	Mice (nb and adult)	Death; Antibody	SM	(9, 24-29)
Gomoka	DAK AR B 2712	01-May-1970	*Anopheles paludis*	Mosquito	Near Bambio on the fourth parallel, Central African Republic	Ungrouped	Mice (nb and adult)	Death; Antibody	SM 16	(30, 31)

Huacho	AR 883	14-Oct-1967	*Ornithodoros amblus*	Tick	Punta Salinas, Huacho, Peru	Kemerovo	Mice (nb)	Sickness	SM 3	(32)
Inini	CAY AN 1093 A	09-Sep-1973	*Pteroglossus aracari*	Bird	Inini (Exper. Station, Pasteur Inst.), French Guiana	Simbu	Mice (nb and adult)	Death; Death; Antibody	P5, SM2, Vero 1	(33, 34)
Jacareacanga	BeAr 295042	11-Nov-1975	Culex sp.	Mosquito	Jacareacanga, Pará, Brazil	Corriparta	Mice (nb)	Death	NH	-
Kasba	IG 15534	19-Jul-1957	*Culex vishnui*	Mosquito	Sathuperi, North Arcot Dist., Tamil Nadu, India	Palyam	Mice (nb and adult); guinea pig (adult)	Sickness and death; Occasional sickness; CF and antibodies detected	SM 2	(35, 36)
Kindia	DAK AR K 502	23-Apr-1983	*Amblyomma variegatum*	Tick	Kindia, Guinea	Palyam	Mice (nb); Mice (adult); guinea pig	Death; Antibody; Antibody	SM 8, Vero 1	(37, 38)
Kununurra	OR 194	15-Apr-1973	*Aedeomyia catasticta*	Mosquito	Kununurra, Western Australia	Ungrouped	Mice (nb and adult)	Paralysis and death; Death; Death	SM 4	(39)
Lake Clarendon	CSIRO 704	03-Jan-1981	*Argas robertsi*	Tick	Gatton, Queensland, Australia	Ungrouped	Mice (nb)	Paralysis and death	SM 6	(30, 40)
Lanjan	TP 94	24-Aug-1960	*Dermacentor auratus*	Tick	Bukit Lanjan, Malaysia	Kaisodi	Mice (nb and adult)	Paralysis and death; Paralysis and death	SM 12	(41-43)
Llano Seco	BFN 3112	23-Jul-1971	*Culex tarsalis*	Mosquito	Llano Seco Ranchero, Butte Co., California, USA	N/A	Mice (nb)	Sickness and death	SM 8	(44, 45)
Marrakai	CSIRO 82 (BH 1857)	01-Oct-1975	*Culicoides schultzei and C. perigrinus*	Mosquito	Beatrice Hill, Northern Territory, Australia	Palyam	Mice (nb)	Paralysis and death	SM 3, Vero 1	(23, 24)
Matariya	EG AN 1477-61	08-Oct-1961	*Sylvia curraca (Lesser whitethroat)*	Bird	West of Port Said, Port Said Gov., Egypt	Matariya	Mice (nb)	Death	SM 6	(46)
Minnal	IG 7481	25-Sep-1956	*Culex vishnui*	Mosquito	Minnal, N. Arcot District, Madras State, India	Ungrouped	Mice (nb); Chick embryos	Death; Death	SM 35	(13, 35, 43)
Mitchell River	MRM 10434	08-Apr-1969	*Culicoides spp.*	Mosquito	Mitchell River, Queensland, Australia	Warrego	Mice (nb and adult)	Paralysis and death; Antibody	SM 2	(9, 27, 28, 47, 48)
Mono Lake	AR 861	18-May-1966	*Argas cooleyi*	Tick	Mono Lake, Mono County, California, USA	Kemerovo	Mice (nb and adult)	Death; Sickness	SM 8	(32)
Monte Dourado	BeAn 385401	8-Aug-1980	*Dasypus novemcinctus*	Armadillos	Almeirim, Pará, Brazil	Changuinola	Mice (nb)	Illness, death	SM 5	(49)
Mykines	Den AR 12	05-Aug-1974	*Ixodes uriae (Ixodes putus)*	Tick	Mykines, Faeroe Islands, Denmark	Kemerove	Mice (nb)	Paralysis and death	SM 3	(50, 51)

Ndelle	DAK AN Y 1444	24-Feb-1974	*Mus minutoides*	Rat	Ndelle, Cameroon	Ungrouped	Mice (nb and adult)	Death; Paralysis and death	SM 9	(52)
Nugget	AUS MI 14847	01-Jan-1972	*Ixodes uriae (Ixodes putus)*	Tick	Macquarie Island, Southern Ocean, Australia	Kemerovo	Mice (nb and adult)	Death; Antibody	SM 6	(53)
Olifantsvlei	SA AR 5133	08-Jan-1963	*Culex pipiens*	Mosquito	Olifantsvlei sewage farm, Johannesburg, South Africa	Olifantsvlei	Mice (nb)	Death	SM 7	(54-57)
Ossa	BT 1820	01-Jan-1961	Human	Human	Almirante, Bocas del Toro Province, Panama	C	Mice (nb); hamster (adult)	Coma, death; Coma, death	P13 (NH), Vero 2	(43, 46, 58-61)
Palestina	76 V 1565	14-May-1975	*Culex paracrybda*	Mosquito	Palestina, Ecuador	Minatitlan	Mice (nb)	Death	SM 3, Vero 2	(62)
Paroo River	GG 668	05-Apr-1973	*Culex annulirostris*	Mosquito	Wanaaring, Paroo River, N.S.W., Australia	Ungrouped	Mice (nb and adult)	Paralysis and death; Antibody	(NH) SM 1	Unk
Porton's	Porton S 1643	1983	*Mansonia uniformis*	Mosquito	Sarawak, Malaysia	Ungrouped	-	-	SM 30	(63)
Seletar	SM 214	16-Jan-1961	*Boophilus microplus*	Tick	Seletar District, Singapore	Kemerovo	Mice (nb)	Illness and death	(NH) SM 3, BHK 1	(64)
Sixgun City	RML 52451	28-Jun-1969	*Argas cooleyi*	Tick	Texas, Randall Co., Sunday Canyon, USA	Kemerovo	Mice (nb)	Death	SM 4	(65, 66)
Timbo	BE AN 41787	27-Apr-1962	*Ameiva ameiva ameiva*	Lizard	Instituto Agronomico do Norte forest, Pará, Brazil	Timbo	Mice (nb and adult)	Death; Antibody	SM 5	(43, 67-69)
Tindholmur	Den AR 2	22-Jul-1974	*Ixodes uriae (Ixodes putus)*	Tick	Tindholmur, Faeroe Islands, Denmark	Kemerovo	Mice (nb)	Paralysis and death	SM 1	(70)
Tsuruse	Mag 271580	04-Aug-1954	*Cyanopica cyanus*	Bird	Tsuruse, Tokyo, Japan	Tete	Mice (nb and adult); hamsters (4-6 wk); emb. eggs (7-8 day)	CNS symptoms; CNS symptoms; CNS symptoms; Hemorrhagic embryos	Vero 1	(71, 72)
Vellore	I 68886	18-Jul-1966	*Culex pseudovishnui* and Culex sp.	Mosquito	Vellore, North Arcot District, Tamil Nadu, India	Palyam	Mice (nb)	Sickness and death	SM 4	(73)
Virgin River	743-366	12-Sep-1974	*Anopheles freeborni*	Mosquito	Beaver Dam Wash, at Virgin River, Arizona	Anopheles A	Mice (nb and adult)	Death; Death	SM 4, Vero 4	(74)
Xingu	BeH388464	Unknwon	Human	Human	Mato Grosso, Brazil	Bunyamwera	-	-	NHH	(75-78)

Yaquina Head	56297-15	19-Aug-1970	*Ixodes uriae (Ixodes putus)*	Tick	Yaquina Head, Lincoln Co., Oregon, USA	Kemerovo	Mice (nb)	Death	SM 7	(79)
Zingilamo	DAK AN B 1245	21-Sep-1970	*Bycanistes sharpe*	Bird	Central African Republic	-	-	-	SM 13	(30)

Legend: SM: SM: Suckling mice; (nb): Newborn; wk: Weeks; P: Passage number; BHK: Baby hamster kidney cells; NH: No passage history; CF: Complement fixation; CNS: Central nervous system symptoms; CPE: Cytopathic effect; N/A: Not available / Not applicable.

Supplementary Table 2. Sequencing status, alignment statistics, and taxonomic classification (family and genus) of the 46 newly sequenced arboviruses in this study.

Family	Genus	Virus	Complete coding sequence	Previous sequencing status	Segment	Average depth coverage
Reoviridae	Orbivirus	Acado	Yes	Complete	1	39380.7
					2	3577.38
					3	4863.35
					4	7322.65
					5	5921.3
					6	13149.8
					7	474.397
					8	1996.06
					9	1380.06
					10	537.45
		Andasibe	Yes	Unsequenced	1	394.515
					2	392.453
					3	5169.5
					4	2177.04
					5	1130.11
					6	566.398
					7	476.007
					8	634.927
					9	711.969
					10	532.63
		Arkonam	Yes	Unsequenced	1	4742.59
					2	8552.21
					3	8909.47
					4	8171.15
					5	24204.9
					6	8292.33
					7	27429.8
					8	20467.8
					9	7890.68
					10	11723.4
		Bauline	Yes	Unsequenced	1	1681.09
					2	2194.46
					3	1263.72
					4	4585.59
					5	1718.26
					6	4573.65
					7	2958.84
					8	7099.04
					9	2485.56
					10	2536.39
		Cape Wrath	Yes	Unsequenced	1	1420.85
					2	1554.82
					3	1106.8
					4	2433.88
					5	6917.82
					6	2080.52
					7	7664.29
					8	3603.53
					9	3114.69
					10	2201.95
		Csrio Village	No	Unsequenced	1	7204.26
					2	10722.9
					3	5674.92
					4	6764.11
					5	22793.8
					6	5224.63
					7	11322.7
					8	12846.5
					9	6067.93
					10	3356.47
		D'Aguilar	No	Partial	1	980.331
					2	1288.32
					3	1322.78

				4	2228.09
				5	4953.14
				6	1249.8
				7	2888.29
				8	3675.61
				9	3849.64
				10	1327.02
	Gomoka	No	Unsequenced	1	232.118
				2	252.408
				3	181.598
				4	1078.98
				5	380.287
				6	348.739
				7	593.163
				8	1228.48
				9	474.06
				10	317.106
	Huacho	No	Unsequenced	1	189.327
				2	173.602
				3	118.937
				4	290.613
				5	522.055
				6	220.547
				7	578.187
				8	246.22
				9	596.333
	Jacareacanga	No	Unsequenced	1	2792.69
				2	2714.28
				3	2389.64
				4	2706.35
				5	2702.97
				6	2705.83
				7	2576.52
				8	2802.08
				9	2482.75
				10	2941.6
	Kasba	Yes	Partial	1	452.62
				2	439.55
				3	516.865
				4	781.229
				5	859.572
				6	1481.98
				7	1700.03
				8	2319.98
				9	1054.96
				10	870.121
	Kindia	Yes	Unsequenced	1	6294.03
				2	9015.76
				3	7550.43
				4	10175.1
				5	34151.9
				6	9393.7
				7	16884.3
				8	17909.5
				9	14887.3
				10	6493.59
	Llano Seco	No	Unsequenced	1	12095.4
				2	3542.08
				3	750.158
				4	11842.5
				5	7966.63
				6	6093.27
				7	5509.99
				8	357.919
				9	1220.95
				10	21745.1
	Marrakai	No	Partial	1	34810.7
				2	21408.6

				3	16745.7
				4	33454.5
				5	60384.9
				6	15469.2
				7	591.104
				8	16181.6
				9	2179.96
	Minnal	Yes	Partial	1	471.509
				2	833.709
				3	651.287
				4	246.508
				5	1656.79
				6	1092.98
				7	790.885
				8	884.305
				9	553.668
				10	1223.7
	Mitchell River	No	Unsequenced	1	6618.21
				2	3861.39
				3	5663.74
				4	15772.3
				5	3230.76
				6	18177.6
				7	3235.15
				8	21683.3
				9	3345.14
				10	3093.29
	Mono Lake	No	Complete	1	102.009
				2	101.613
				3	696.394
				4	149.612
				5	317.845
				6	147.143
				7	303.511
				8	141.181
				9	224.24
				10	160.228
	Monte Dourado	No	Unsequenced	1	13.78
				2	9.72
				3	15.82
				4	40.98
				5	33.26
				6	76.6
				7	48.96
				8	115.06
				9	129.41
				10	87.58
	Mykines	No	Unsequenced	1	262.811
				2	239.914
				3	235.93
				4	922.716
				5	536.022
				6	468.6
				7	1250.45
				8	1485.15
				9	800.424
				10	1135.28
	Nugget	No	Partial	1	1144.6
				2	1868.37
				3	567.834
				4	5351.99
				5	2126
				6	1548.96
				7	4725.8
				8	2533.8
				9	1281.2
				10	1667.68
	Paroo River	Yes	Partial	1	832.376

					2	165.874
					3	124.93
					4	213.104
					5	383.022
					6	114.075
					7	164.835
					8	455.614
					9	281.334
					10	350.642
		Seletar	No	Unsequenced	1	1119.6
					2	1743.47
					3	1914.32
					4	1940.02
					5	4169.25
					6	1637.5
					7	6079.9
					8	2128.45
					9	4004.8
					10	2420.21
		Sixgun City	No	Unsequenced	1	156.608
					2	218.546
					3	183.542
					4	486.04
					5	854.704
					6	434.878
					7	571.117
					8	286.35
					9	536.412
					10	349.904
		Tindholmur	Yes	Unsequenced	1	157.787
					2	232.178
					3	132.34
					4	494.507
					5	436.753
					6	324.1
					7	323.087
					8	1094.55
					9	296.461
					10	652.544
		Vellore	No	Partial	1	641.375
					2	115.244
					3	991.823
					4	120.764
					5	926.244
					6	163.824
					7	271.776
					8	274.668
					9	212.423
					10	174.561
		Yaquina Head	No	Unsequenced	1	435.379
					2	354.216
					3	277.164
					4	749.936
					5	614.027
					6	856.771
					7	777.268
					8	184.273
					9	132.748
					10	809.637
Reoviridae	Orthoreovirus	Ndelle	No	Partial	1	14699.4
					2	13158
					3	16720.2
					4	13963.9
					5	11714.2
					6	33003.2
					7	11654.8
					8	19016.9
					9	23793.1

					10	23645.4
Peribunyaviridae	Orthobunyavirus	Bobia	Yes	Unsequenced	L	3930.59
					M	11036.5
					S	21997.8
		Botambi	Yes	Complete	L	5954.99
					M	8331.28
					S	28226.3
		Brus Laguna	Yes	Unsequenced	L	1692.93
					M	3429.55
					S	8156.07
		Inini	Yes	Complete	L	36505
					M	72306.7
					S	122289
		Kununurra	Yes	Unsequenced	L	9847.55
					M	12790.4
					S	71092.2
		Olifantsvlei	No	Unsequenced	L	919.522
					M	2940.87
					S	7658.21
		Ossa	Yes	Complete	L	12179.5
					M	37703.2
					S	103927
		Palestina	Yes	Unsequenced	L	27926.6
					M	31962.9
					S	106023
		Tsuruse	Yes	Unsequenced	L	2211.8
					M	8079.26
					S	32266.8
		Virgin River	Yes	Unsequenced	L	4219.86
					M	6198.28
					S	30194.3
		Xingu	No	Partial	L	508.28
					M	663.78
					S	3556.58
Peribunyaviridae	Pacuvirus	Arboledas	Yes	Unsequenced	L	29116.6
					M	72586.6
					S	253791
Phenuiviridae	Uukuvirus	Lanjan	Yes	Partial	L	8214.53
					M	14592.9
					S	11842.3
Nairoviridae	Orthonairovirus	Lake Clarendon	Yes	Unsequenced	L	202.478
					M	351.773
					S	494.457
Rhabdoviridae	Hapavirus	Porton's	Yes	Partial	N/A	18330.2
	Sripuvirus	Timbo	Yes	Partial	N/A	6087.22
	Sunrhavirus	Matariya	Yes	Complete	N/A	7770.29
Togaviridae	Alphavirus	Zingilamo	Yes	Complete	N/A	98811.6
Flaviviridae	Orthoflavivirus	Cowbone Ridge	Yes	Partial	N/A	5280.47

Legend: **N/A**: Not available / Not applicable.

Supplementary Table 3. Predicted signal peptides in structural glycoproteins and polyproteins of arboviruses analyzed in this study (80).

Virus	Cleavage site	Signal Peptide (Sec/SPI)
Lanjan	Between 18-19	0.9988
Lake Clarendon	Between 16-17	0.9992
Arboledas	Between 16-17	0.9992
Virgin River	Between 15-16	0.9992
Tsuruse	Between 15-16	0.999
Palestina	Between 16-17	0.9991
Olifantsvlei	Between 23-24	0.9992
Ossa	Between 22-23	0.9991

Kununurra	Between 15-16	0.9993
Brus Laguna	Between 14-15	0.9988
Botambi	Between 20-21	0.9992
Bobia	Between 23-24	0.9992
Timbo	Between 19-20	0.9992
Matariya	Between 19-20	0.9058

Supplementary Table 4. Genomic characterization of arboviruses analyzed in this study.

Family	Genus	Virus	Genome segment	Genome size (nt)	Encoded proteins	Encoded proteins position	GenBank ID
Reoviridae	Orbivirus	Acado	1	3,915	RNA-dependent RNA polymerase (VP1)	10-3,876	PV804376
			2	2,794	VP2	<1-2,737	PV804378
			3	2,310	VP3(T2)	35-2,251	PV804379
			4	2,034	VP4(Cap)	16-1,947	PV804380
			5	1,791	NS1(TuP)	7-1,776	PV804381
			6	1,678	VP5	53-1,636	PV804382
			7	1,169	VP7(T13)	10-1,074	PV804383
			8	1,194	NS2(ViP)	41-1,150	PV804384
			9	1,129	VP6(Hel)VP6a and NS4	45-1,085 and 139-600	PV804385
			10	763	NS3	4-720	PV804377
		Andasibe	1	3,961	RNA-dependent RNA polymerase (VP1)	24-3,938	PV804367
			2	3,042	VP2	13-2,946	PV804369
			3	2,791	VP3(T2)	25-2,718	PV804370
			4	1,973	VP4(Cap)	12-1,961	PV804371
			5	1,800	NS1(TuP)	29-1,678	PV804372
			6	1,631	VP5	21-1,607	PV804330
			7	1,163	VP7(T13)	1-1,044	PV804373
			8	655	NS2(ViP)	41->655	PV804374
			9	899	VP6(Hel)VP6a	40-861	PV804375
			10	835	NS3	43-738	PV804368
		Arkonam	1	4,042	RNA-dependent RNA polymerase (VP1)	4-3,981	PV804575
			2	2,393	VP2	19-2,352	PV804577
			3	2,858	VP3(T2)	49-2,808	PV804578
			4	1,996	VP4(Cap)	9-1,943	PV804579
			5	1,779	NS1(TuP)	32-1,720	PV804580
			6	1,661	VP5	37-1,605	PV804581
			7	1,190	VP7(T13)	19-1,074	PV804582
			8	1,205	NS2(ViP)	58-1,158	PV804583
			9	933	VP6(Hel)VP6a	18-881	PV804584
			10	774	NS3	35-619	PV804576
		Bauline	1	3,897	RNA-dependent RNA polymerase (VP1)	12-3,869	PV804421
			2	2,794	VP2	21-2,747	PV804423
			3	1,939	VP3(T2)	11-1,918	PV804424
			4	1,751	VP4(Cap)	38-1,699	PV804425
			5	1,731	NS1(TuP)	41-1,636	PV804426
			6	1,666	VP5	21-1,634	PV804427
			7	1,179	VP7(T13)	18-1,091	PV804428
			8	1,210	NS2(ViP)	46-1,164	PV804429
			9	1,055	VP6(Hel)VP6a and NS4	54-1,019 and 175-747	PV804430
			10	705	NS3	19-663	PV804422
		Cape Wrath	1	3,934	RNA-dependent RNA polymerase (VP1)	32-3,889	PV804338
			2	2,790	VP2	12-2,738	PV804340

		3	1,952	VP3(T2)	28-1,935	PV804341
		4	1,739	VP4(Cap)	13-1,692	PV804342
		5	1,772	NS1(TuP)	67-1,662	PV804343
		6	1,682	VP5	26-1,639	PV804344
		7	1,203	VP7(T13)	42-1,115	PV804345
		8	1,239	NS2(ViP)	70-1,188	PV804346
		9	1,073	VP6(Hel)VP6a and NS4	66-1,031 and 187-759	PV804347
		10	716	NS3	32-676	PV804339
	Csrio Village	1	3,927	RNA-dependent RNA polymerase (VP1)	23-3,910	PV804553
		2	3,075	VP2	37-3,027	PV804555
		3	2,759	VP3(T2)	8-2,722	PV804556
		4	1,962	VP4(Cap)	<1-1,919	PV804557
		5	1,778	NS1(TuP)	32-1,669	PV804558
		6	1,606	VP5	21-1,586	PV804559
		7	1,155	VP7(T13)	19-1,065	PV804560
		8	1,056	NS2(ViP)	22-1,023	PV804561
		9	877	VP6(Hel)VP6a and NS4	20-838 and 144-395	PV804562
		10	724	NS3	17-652	PV804554
	D'Aguilar	1	3,924	RNA-dependent RNA polymerase (VP1)	20-3,907	PV804477
		2	3,029	VP2	27-3,005	PV804479
		3	2,770	VP3(T2)	14-2,728	PV804480
		4	1,972	VP4(Cap)	<1-1922	PV804481
		5	1,772	NS1(TuP)	36-1,673	PV804482
		6	1,618	VP5	32-1,597	PV804483
		7	1,153	VP7(T13)	18-1,064	PV804484
		8	1,069	NS2(ViP)	27-1,034	PV804485
		9	880	VP6(Hel)VP6a and NS4	20-838 and 144-395	PV804486
		10	730	NS3	1-657	PV804478
	Gomoka	1	3,881	RNA-dependent RNA polymerase (VP1)	<1-3,854	PV804325
		2	2,794	VP2	19-2,745	PV804545
		3	1,936	VP3(T2)	6-1,913	PV804546
		4	1,687	VP4(Cap)	4-1,683	PV804547
		5	1,731	NS1(TuP)	41-1,636	PV804548
		6	1,666	VP5	21-1,634	PV804549
		7	1,181	VP7(T13)	18-1,091	PV804550
		8	1,195	NS2(ViP)	35-1,153	PV804551
		9	1,046	VP6(Hel)VP6a and NS4	45-1,010 and 166-738	PV804552
		10	701	NS3	18-662	PV804544
	Huacho	1	3,875	RNA-dependent RNA polymerase (VP1)	<1-3,857	PV804498
		2	2,790	VP2	<1-2,744	PV804499
		3	1,926	VP3(T2)	<1-1,903	PV804500
		4	1,786	VP4(Cap)	53-1,750	PV804501
		5	1,698	NS1(TuP)	32-1,642	PV804502
		6	1,485	VP5	<1-1,451	PV804503
		7	1,173	VP7(T13)	18-1,088	PV804504
		8	1,206	NS2(ViP)	28-1,167	PV804505
		9	1,010	VP6(Hel)VP6a and NS4	6-953 and 79-684	PV804506
	Jacareacanga	1	3,891	RNA-dependent RNA polymerase (VP1)	<1-3,851	PV804618
		2	2,773	VP2	<1-2,735	PV804620
		3	2,274	VP3(T2)	<1-2,237	PV804621
		4	2,010	VP4(Cap)	<1-1928	PV804622
		5	1,212	NS1(TuP)	<1->1,212	PV804623
		6	1,666	VP5	40-1,623	PV804624
		7	1,118	VP7(T13)	<1-1,057	PV804626

	8	1,162	NS2(ViP)	<1-1,100	PV804625
	9	1,130	VP6(Hel)VP6a and NS4	48-1085 and 142-600	PV804627
	10	751	NS3	1-712	PV804619
Kasba	1	3,918	RNA-dependent RNA polymerase (VP1)	15-3,902	PV804598
	2	3,059	VP2	1-3,021	PV804600
	3	2,778	VP3(T2)	7-2,721	PV804601
	4	1,965	VP4(Cap)	11-1,933	PV804602
	5	1,757	NS1(TuP)	36-1,673	PV804603
	6	1,611	VP5	25-1,590	PV804604
	7	1,152	VP7(T13)	18-1,064	PV804605
	8	1,053	NS2(ViP)	19-1,020	PV804606
	9	874	VP6(Hel)VP6a	20-838	PV804607
	10	725	NS3	19-654	PV804599
Kindia	1	3,930	RNA-dependent RNA polymerase (VP1)	23-3,910	PV804452
	2	3,073	VP2	17-3,046	PV804454
	3	2,771	VP3(T2)	14-2,725	PV804455
	4	1,940	VP4(Cap)	1-1,923	PV804456
	5	1,771	NS1(TuP)	36-1,673	PV804457
	6	1,603	VP5	20-1,585	PV804458
	7	1,154	VP7(T13)	19-1,065	PV804459
	8	1,055	NS2(ViP)	20-1,018	PV804460
	9	877	VP6(Hel)VP6a and NS4	20-838 and 144-395	PV804461
	10	728	NS3	19-654	PV804453
Llano Seco	1	3,924	RNA-dependent RNA polymerase (VP1)	16-3,915	PV804534
	2	2,802	VP2	27-2,744	PV804536
	3	1,980	VP3(T2)	<1-1,937	PV804537
	4	2,030	VP4(Cap)	26-1,972	PV804538
	5	2,004	NS1(TuP)	23-1,765	PV804539
	6	1,461	VP5	<1-1,402	PV804540
	7	1,168	VP7(T13)	18-1,073	PV804541
	8	1,355	NS2(ViP)	80-1,303	PV804542
	9	1,102	VP6(Hel)VP6a	44-1,069	PV804543
	10	810	NS3	17-810	PV804535
Marrakai	1	3,935	RNA-dependent RNA polymerase (VP1)	23-3,910	PV804360
	2	3,072	VP2	17-3,046	PV804361
	3	2,768	VP3(T2)	14-2,728	PV804362
	4	1,969	VP4(Cap)	15-1,937	PV804363
	5	1,771	NS1(TuP)	40-1,677	PV804358
	6	1,624	VP5	25-1,590	PV804364
	7	1,148	VP7(T13)	19-1,065	PV804366
	8	1,021	NS2(ViP)	17-1,018	PV804359
	9	867	VP6(Hel)VP6a and NS4	9-827 and 133-384	PV804365
Minnal	1	3,917	RNA-dependent RNA polymerase (VP1)	2-3,901	PV804431
	2	2,796	VP2	24-2,741	PV804433
	3	2,397	VP3(T2)	16-2,340	PV804434
	4	2,007	VP4(Cap)	2-1,948	PV804435
	5	1,794	NS1(TuP)	29-1,771	PV804436
	6	1,678	VP5	28-1,617	PV804437
	7	1,180	VP7(T13)	11-1,066	PV804438
	8	1,327	NS2(ViP)	54-1,274	PV804439
	9	1,096	VP6(Hel)VP6a	36-1,061	PV804440
	10	885	NS3	16-807	PV804432
Mitchell River	1	3,979	RNA-dependent RNA polymerase (VP1)	21-3,950	PV804608
	2	1,972	VP2	<1-1,922	PV804610

		3	2,773	VP3(T2)	<1-2,726	PV804611
		4	1,994	VP4(Cap)	34-1,959	PV804612
		5	1,752	NS1(TuP)	39-1,691	PV804613
		6	1,644	VP5	45-1,631	PV804614
		7	1,159	VP7(T13)	18-1,070	PV804615
		8	1,151	NS2(ViP)	13-1,095	PV804616
		9	1,112	VP6(Hel)VP6a	1-1,062	PV804617
		10	860	NS3	23-781	PV804609
	Mono Lake	1	3,856	RNA-dependent RNA polymerase (VP1)	<1-3,848	PV804386
		2	2,760	VP2	<1-2,722	PV804388
		3	1,916	VP3(T2)	<1-1,897	PV804389
		4	1,727	VP4(Cap)	1-1,698	PV804390
		5	1,773	NS1(TuP)	26-1,711	PV804391
		6	1,658	VP5	22-1,629	PV804392
		7	1,170	VP7(T13)	8-1,078	PV804393
		8	1,187	NS2(ViP)	16-1,152	PV804394
		9	990	VP6(Hel)VP6a and NS4	4-951 and 77-676	PV804395
		10	723	NS3	10-678	PV804387
	Monte Dourado	1	2,377	RNA-dependent RNA polymerase (VP1)	<1->2,377	PV804628
		2	3,398	VP2	57-3,395	PV804630
		3	2,692	VP3(T2)	81-2,675	PV804631
		4	1,944	VP4(Cap)	<1-1,901	PV804632
		5	1,693	NS1(TuP)	18-1,691	PV804633
		6	1,612	VP5	7-1,593	PV804634
		7	1,072	VP7(T13)	<1-1,049	PV804635
		8	976	NS2(ViP)	8-964	PV804636
		9	832	VP6(Hel)VP6a and NS4	8-832 and 126-389	PV804637
		10	672	NS3	48-671	PV804629
	Mykines	1	3,933	RNA-dependent RNA polymerase (VP1)	49-3,906	PV804524
		2	2,788	VP2	11-2,737	PV804526
		3	1,918	VP3(T2)	<1-1,903	PV804527
		4	1,708	VP4(Cap)	<1-1,658	PV804528
		5	1,755	NS1(TuP)	59-1,654	PV804529
		6	1,657	VP5	10-1,623	PV804530
		7	1,186	VP7(T13)	19-1,092	PV804531
		8	1,234	NS2(ViP)	48-1,166	PV804532
		9	1,056	VP6(Hel)VP6a and NS4	55-1,020 and 176-748	PV804533
		10	705	NS3	19-663	PV804525
	Nugget	1	3,890	RNA-dependent RNA polymerase (VP1)	<1-3,870	PV804396
		2	2,773	VP2	3-2,729	PV804398
		3	1,947	VP3(T2)	<1-1,921	PV804399
		4	1,731	VP4(Cap)	7-1,674	PV804400
		5	1,702	NS1(TuP)	27-1,622	PV804401
		6	1,662	VP5	22-1,635	PV804402
		7	1,161	VP7(T13)	1-1,074	PV804403
		8	1,178	NS2(ViP)	29-1,147	PV804404
		9	1,048	VP6(Hel)VP6a and NS4	31-1,017 and 143-745	PV804405
		10	689	NS3	6-650	PV804397
	Paroo River	1	3,923	RNA-dependent RNA polymerase (VP1)	16-3,900	PV804441
		2	2,825	VP2	12-2,732	PV804443
		3	2,256	VP3(T2)	4-2,244	PV804444
		4	2,029	VP4(Cap)	17-1,966	PV804445
		4	2,085	NS1(TuP)	44-1,810	PV804446
		6	1,653	VP5	24-1,616	PV804447
		7	1,118	VP7(T13)	22-1,074	PV804448

			8	1,103	NS2(ViP)	30-1,016	PV804449
			9	943	VP6(Hel)VP6a	28-927	PV804450
			10	720	NS3	1-657	PV804442
		Seletar	1	3,936	RNA-dependent RNA polymerase (VP1)	<1-3,908	PV804471
			2	2,810	VP2	36-2,765	PV804336
			3	1,903	VP3(T2)	<1-1,862	PV804335
			4	1,819	VP4(Cap)	38-1,780	PV804334
			5	1,782	NS1(TuP)	49-1,656	PV804473
			6	1,678	VP5	5-1,633	PV804333
			7	1,170	VP7(T13)	6-1,070	PV804474
			8	1,186	NS2(ViP)	12-1,145	PV804475
			9	986	VP6(Hel)VP6a	1-942	PV804476
			10	717	NS3	4-663	PV804472
		Sixgun City	1	3,862	RNA-dependent RNA polymerase (VP1)	<1-3,850	PV804408
			2	2,637	VP2	<1-2,579	PV804410
			3	1,877	VP3(T2)	<1-1,868	PV804411
			4	1,739	VP4(Cap)	8-1,705	PV804412
			5	1,697	NS1(TuP)	31-1,641	PV804413
			6	1,649	VP5	5-1,612	PV804414
			7	1,158	VP7(T13)	4-1,074	PV804415
			8	1,193	NS2(ViP)	19-1155	PV804416
			9	985	VP6(Hel)VP6a and NS4	1-947 and 73-672	PV804417
			10	727	NS3	10-678	PV804409
		Tindholmur	1	3,894	RNA-dependent RNA polymerase (VP1)	9-3,866	PV804514
			2	2,788	VP2	19-2,745	PV804516
			3	1,917	VP3(T2)	<1-1,902	PV804517
			4	1,733	VP4(Cap)	26-1,687	PV804518
			5	1,734	NS1(TuP)	42-1,637	PV804519
			6	1,666	VP5	23-1,636	PV804520
			7	1,191	VP7(T13)	32-1,105	PV804521
			8	1,200	NS2(ViP)	39-1,157	PV804522
			9	1,043	VP6(Hel)VP6a and NS4	44-1,009 and 165-737	PV804523
			10	722	NS3	38-682	PV804515
		Vellore	1	3,913	RNA-dependent RNA polymerase (VP1)	9-3,896	PV804488
			2	3,056	VP2	18-3,032	PV804490
			3	2,753	VP3(T2)	69-2,711	PV804491
			4	1,954	VP4(Cap)	<1-1,918	PV804492
			5	1,731	NS1(TuP)	41-1,678	PV804493
			6	1,579	VP5	1-1,560	PV804494
			7	1,144	VP7(T13)	11-1,057	PV804495
			8	1,049	NS2(ViP)	10-1,008	PV804496
			9	903	VP6(Hel)VP6a and NS4	49-867 and 173-424	PV804497
			10	731	NS3	2-658	PV804489
		Yaquina Head	1	3,761	RNA-dependent RNA polymerase (VP1)	<1-3,736	PV804462
			2	2,773	VP2	<1-2,725	PV804464
			3	1,446	VP3(T2)	<1-1,436	PV804337
			4	1,717	VP4(Cap)	9-1,670	PV804465
			5	1,727	NS1(TuP)	40-1,635	PV804466
			6	1,639	VP5	4-1,617	PV804467
			7	1,164	VP7(T13)	4-1,077	PV804468
			8	1,185	NS2(ViP)	23-1,141	PV804469
			9	1,041	VP6(Hel)VP6a and NS4	41-1,006 and 162-734	PV804470
			10	689	NS3	8-652	PV804463
Reoviridae	Orthoreovirus	Ndelle	1	3,834	Lamba-3 protein (RdRP)	5-3,808	PV804332

			2	3,914	Lamba-2 protein	5-3,874	PV804563
			3	3,893	Lamda-1 protein	1-3,841	PV804564
			4	2,307	Mu-2 protein	9-2,219	PV804565
			5	2,181	Mu-1 protein	19-2,145	PV804566
			6	2,241	Mu-NS protein	12-2,177	PV804567
			7	1,400	Sigma-1 protein	6-1,373	PV804568
			8	1,322	Sigma-2 protein	16-1,272	PV804569
			9	1,175	Sigma-NS protein	17-1,117	PV804570
			10	1,189	Sigma-3 protein	26-1,123	PV804571
Peribunyaviridae	Orthobunyavirus	Bobia	L	7,007	RNA-dependent RNA polymerase	72-6,887	PV804352
			M	4,718	Polyprotein	63-4,496	PV804353
			S	1,228	Structural nucleocapsid and nonstructural proteins	108-812 and 181-384	PV804354
		Botambi	L	6,814	RNA-dependent RNA polymerase	44->6,814	PV804331
			M	4,666	Polyprotein	49-4,455	PV804406
			S	1,096	Structural nucleocapsid and nonstructural proteins	78-782 and 103-354	PV804407
		Brus Laguna	L	6,935	RNA-dependent RNA polymerase	39-6,860	PV804349
			M	5,000	Polyprotein	34-4,812	PV804350
			S	1,184	Structural nucleocapsid and nonstructural proteins	79-795 and 107-499	PV804351
		Inini	L	6,854	RNA-dependent RNA polymerase	39-6,824	PV804355
			M	4,437	Polyprotein	7-4,293	PV804356
			S	964	Structural nucleocapsid and nonstructural proteins	39-740 and 61-351	PV804357
		Kununurra	L	6,959	RNA-dependent RNA polymerase	64-6,864	PV804594
			M	4,990	Polyprotein	139-4,809	PV804595
			S	997	Structural nucleocapsid and nonstructural proteins	63-815 and 124-432	PV804596
		Olifantsvlei	L	6,772	RNA-dependent RNA polymerase	1-6,658	PV804508
			M	4,705	Polyprotein	45-4,478	PV804509
			S	1,136	Structural nucleocapsid and nonstructural proteins	94-798 and 167-370	PV804510
		Ossa	L	7,129	RNA-dependent RNA polymerase	43-6,789	PV804329
			M	4,642	Polyprotein	65-4,351	PV804328
			S	1,169	Structural nucleocapsid and nonstructural proteins	88-795 and 50-367	PV804487
		Palestina	L	6,997	RNA-dependent RNA polymerase	56-6,814	PV804585
			M	4,800	Polyprotein	83-4,417	PV804586
			S	1,201	Structural protein	94-804	PV804587

		Tsuruse	L	7,038	RNA-dependent RNA polymerase	69-6,911	PV804591
			M	4,458	Polyprotein	45-4,346	PV804592
			S	1,027	Structural protein	67-843	PV804593
		Virgin River	L	6,848	RNA-dependent RNA polymerase	33-6,755	PV804511
			M	4,486	Polyprotein	54-4,331	PV804512
			S	988	Structural protein	61-798	PV804513
		Xingu	L	6,759	RNA-dependent RNA polymerase	46-6,759	PV804638
			M	4,250	Polyprotein	62-4,249	PV804326
			S	836	Structural nucleocapsid and nonstructural proteins	8-709 and 27-332	PV804639
Peribunyaviridae	Pacuvirus	Arboledas	L	6,887	RNA-dependent RNA polymerase	68-6,763	PV804572
			M	4,633	Polyprotein	40-4,398	PV804573
			S	973	Structural protein	72-803	PV804574
Phenuiviridae	Uukuvirus	Lanjan	L	6,526	RNA-dependent RNA polymerase	41-6,406	PV804588
			M	3,269	Polyprotein	46-3,081	PV804589
			S	1,725	Structural nucleocapsid and nonstructural proteins	58-804 and 1,703-894	PV804590
Nairoviridae	Orthonairovirus	Lake Clarendon	L	12,135	Large	53-11,899	PV804418
			M	4,578	Glycoprotein	4-4,344	PV804419
			S	1,786	Nucleocapsid	58-1,557	PV804420
Rhabdoviridae	Hapavirus	Porton's	-	12,044	Nucleoprotein, phosphoprotein, U1 putative protein, matrix, glycoprotein, U2 putative protein, U3 putative protein, polymerase	48-1,355, 1,395-2,147, 2,171-2,662, 2,686-3,372, 3,403-5,286, 4,196-4,435, 5,310-5,657, 5,681-12,044	PV804348
	Sripuvirus	Timbo	-	11,240	Nucleoprotein, U1 putative protein, phosphoprotein, matrix, Mx putative protein, glycoprotein, Gx putative protein, polymerase	1-1,185, 1,258-1,599, 1,659-2,399, 2,438-3,016, 3,013-3,252, 3,284-4,813, 3,294-3,578, 4,847-11,143	PV804451
	Sunrhavirus	Matariya	-	10,844	Nucleoprotein, U1 putative protein, phosphoprotein, matrix, U2 putative protein, glycoprotein, polymerase	<1-1,252, 1,200-2,045, 1,324-1,626, 2,021-2,584, 2,602-2,817, 2,843-4,540, 4,543-10,779	PV804327
Togaviridae	Alphavirus	Cowbone Ridge	-	10,641	Polyprotein	90-10,217	PV804507
Flaviviridae	Orthoflavivirus	Zingilamo	-	11,633	Structural nucleocapsid and nonstructural proteins (nsP1 and nsP2)	7,432-11,193, 92-5,530 and 5,651-7,393	PV804597

Supplementary Table 5. Genome sequences used in the phylogenetic analyses.

Family/subfamily	Genus	Virus name	Abbreviation	GenBank ID
Sedoreoviridae	*Orbivirus*	Great Island	GIV	YP3896059

		Broadhaven	BRDV	P35934
		Lipovnik	LIPV	ADM88604
		Tribec	TRBV	ADM88607
		Kemerovo	KEMV	ADM88610
		Chenuda	CNUV	YP9158879
		Wad Medani	WMV	YP9158883
		Chobar Gorge	CGV	YP9158902
		Yunnan	YOUV	YP443926
		Middle Point	MPOV	ABU95015
		Guangxi	GXOV	AXS77999
		Peruvian horse sickness	PHSV	YP460039
		Mobuck	MBV	YP8719913
		Sathuvachari	SVIV	AGE32262
		Wongorr mm13443	WGRV-mrm13443	AAB47240
		Wongorr v199	WGRV-V199	AAB47239
		Wongorr v1447	WGRV-V1447	AAB47237
		Wongorr v195	V595	AAB47238
		Palyam	PALV	YP52934
		Umatilla	UMAV	AEE98369
		Corriparta	CORV	AGT51055
		Epizootic hemorrhagic disease 7	EHDV-7	CAN89141
		Epizootic hemorrhagic disease 6	EHDV-6	CAN89132
		Epizootic hemorrhagic disease 1	EHDV-1	CAN89079
		Bluetongue 8	BTV-8	CAM57244
		Bluetongue 1	BTV-1	ABA60773
		Corsican bluetongue	BTV-2	AAM94161
		Pata	PATAV	AFH41521
		Tibet	TIBOV	APT68076
		Tilligerry	TILV	AFH41501
		Eubenangee	EUBV	AFH41511
		Wallal	WALV	AIT55704
		Changuinola	CGLV	AFX73367
		Warrego	WARV	AIT55715
		African horse sickness 6	AHSV-6	AAC40995
		African horse sickness 1	AHSV-1	CAP04842
		African horse sickness 2	AHSV-02-07	ACI41992
		Orungo	ORUV	AFX73389
		Lebombo	LEBV	AFX73377
		Equine encephalosis	EEV	YP9507688
		St Croix River	SCRV	YP52943
Spinoreoviridae	*Orthoreovirus*	Piscine orthoreovirus	PRV	KY429952
		Mammalian orthoreovirus 2	MRV-2	JX204746
		Mammalian orthoreovirus 3	MRV-3	EF494444
		Mammalian orthoreovirus 1	MRV-1	KX263316
		Reptilian orthoreovirus	RRV-47/02	KC852162
		Reptilian orthoreovirus	RRV-CH1197/96	NC_078116
		Baboon orthoreovirus	BRV	NC_015885
		Mahlapitsi	MAHLV	NC_029920
		Broome	BrRV	NC_014245
		Muscovy duck reovirus S14	MdRVN-S14	DQ989557
		Goose reovirus D20/99	MdRV-goC	KF809671
		Muscovy duck reovirus J18	MdRV-N	JX478268
		Goose orthoreovirus 03G	MdRV-goN	JX145337
		Avian orthoreovirus 176	ARV176	KF741715
		Avian orthoreovirus TX99	ARV-TX99	AY444910
		Nelson Bay orthoreovirus - Pulau	NBV-Pulau	NC_038658
		Nelson Bay orthoreovirus - Sikamat	NBV-Sikamat	JF811583
		Nelson Bay orthoreovirus	NBV	AF059722
		Avian orthoreovirus Corvid	ARV-Corvid	LC579560
		Avian orthoreovirus Psittaciform	ARV-Psittaciform	AB914769
		Pycnonotidae	PyRV	AB914769

Peribunyaviridae	*Orthobunyavirus*	Abras	ABRV	MH017275
		Babahoya	BABV	MH017276
		Patois	PATV	MH017277
		Patois	PATV	OP263038
		Shark River	SRV	MH017278
		Bellavista	BELLV	KX161718
		Resistencia	RTAV	MK896480
		Antequera	ANTV	MK896641
		Acara	ACAV	MK896653
		Benevides	BVSV; BENV	MK896620
		Benfica	BENV; BNFV	MK896617
		Ananindeua	ANUV	MG821226
		Guama	GMAV	KP792666
		Bimiti	BIMV	KP792657
		Moju	MOJUV	KP792675
		Catu	CATUV	KP792660
		Mahogany hammock	MHV	KP835520
		Timboteua	TBTV	MK896447
		Bushbush	BSBV	MK896599
		Moriche	MORV	MK896521
		Juan Diaz	JDV	MK896554
		Capim	CAPV	KT160026
		Guajara	GJAV	KP792663
		Bertioga	BERV	MK896614
		Cananeia	CNAV	MK896591
		Guaratuba	GTBV	MK896570
		Itimirim	ITIV	MK896557
		Mirim	MIRV	KY013487
		Minatitlan	MNTV	MK896527
		Apeu	APEUV	MG029269
		Marituba	MTBV	KF254770
		Oriboca	ORIV	KF254773
		Murutucu	MURV	MK896518
		Restan	RESV	MK896477
		Gumbo Limbo	GLV	MK896567
		Nepuyo	NEPV	MG029287
		Bruconha	BRUV	KM280929
		Caraparu	CARV	KF254776
		Itaqui	ITQV	MG029275
		Itaya	ITYV	KM092512
		Madrid	MADV	KF254779
		Vinces	VINV	MK896441
		Enseada	ENSV	KU178983
		Belem	BLMV	MK896623
		Brazoran	BRAZV	KC854418
		Pacora	PCAV	MK896500
		Aino	AINOV	HE795087
		Shuni	SHUV	KF153118
		Peaton	PEAV	HE795093
		Sango	SANV	HE795099
		Simbu	SIMV	HE795108
		Douglas	DOUV	HE795090
		Sathuperi	SATV	HE795102
		Schmallenberg	SBV	KC355457
		Shamonda	SHAV	HE795105
		Akabane	AKAV	AB190458
		Tinaroo	TINV	MH484339
		Yaba-7	Y7V	MH484348
		Sabo	SABOV	HE795096
		Balagodu	BLGV	MH507154
		Ingwavuma	INGV	KF697139
		Mermet	MERV	KF697153
		Cat Que	CQV	JQ675598
		Oya	OYAV	JX983194
		Manzanilla	MANV	KF697150
		Buttonwillow	BUTV	KF697160
		Thimiri	THIV	MH484336
		Taniyama	unavailable	LC698004

	Bobaya	BOBV	MW415981
	Facey's Paddock	FPV	KF697138
	Utinga	UTIV	KF697154
	Jatobal	JATV	JQ675603
	Oropouche	OROV	AF484424
	Iquitos	IQTV	KF697142
	Perdoes	PDEV	KP691627
	Belmont	BELV	OR077279
	Parker's Farm	PFV	OR077285
	Leanyer	LEAV	HM627178
	Sedlec	SEDV	MH484327
	Batama	BMAV	MK896626
	Tete	TETEV	KP792681
	I612045	unavailable	HM627179
	Matruh	MTRV	KP792693
	Bahig	BAHV	KP792654
	Lichuan	LICV	MT198373
	Oyo	OYOV	HM639780
	Ness Ziona	NZV	MH018032
	Anadyr	ANADV	KU159766
	Cache Valley	CVV	KX100135
	Tlacotalpan	TALV	KX100120
	Potosi	POTV	MF066368
	Fort Sherman	FSV	KX100132
	Laguna Larga	LLV	KX100111
	Tensaw	TENV	FJ943510
	Maguari	MAGV	KY910429
	Playas	PLAV	KX100129
	Northway	NORV	MH484312
	Main Drain	MDV	MH484306
	Lokern	LOKV	MG828823
	Santa Rosa	SARV	MH484324
	Batai	BATV	JX846606
	Bozo	BOZOV	MH370824
	Bunyamwera	BUNV	X14383
	Ngari	NRIV	KC608152
	Ilesha	ILEV	KF234075
	Shokwe	SHOV	MH484330
	Birao	BIRV	MH370821
	Ebinur lake	EBIV	KJ710425
	Anhembi	AMBV	JN572062
	Iaco	IACOV	JN572065
	Sororoca	SORV	JN572071
	Cachoeira Porteira	CPOV	JN968590
	Macaua	MCAV	JN572068
	Wyeomyia	WYOV	JN572080
	Taiassui	TAIAV	JN572074
	Tucunduba	TUCV	KX579499
	Kairi	KRIV	KR260738
	Guaroa	GROV	KM245524
	Bwamba	BWAV	KJ867184
	Pongola	PGAV	KJ867178
	California encephalitis	CEV	KX817312
	La Crosse	LACV	EF485032
	Snowshoe hare	SSHV	EU203678
	Khatanga	KHATV	HQ734817
	Lumbo	LUMV	KX817324
	Tahyna	TAHV	EU665255
	San Angelo	SAV	KX817330
	Jamestown Canyon	JCV	HM007352
	Jerry Slough	JSV	KX817318
	Inkoo	INKV	EU789573
	South River	SORV	KX817336
	Keystone	KEYV	KX817321
	Serra do Navio	SDNV	KX817333
	Melao	MELV	KX817327
	Infirmatus	INFV	KY569262
	Trivittatus	TVTV	KR149249

		Kaeng Khoi	KKV	KJ867205
		Wolkberg	WBV	KX470551
		Mojui dos Campos	MDCV	KJ867202
		Nyando	NDV	KJ867190
		Kowanyama	KOWV	KT82020
		Termeil	TERV	MK896450
		Yacaaba	YACV	OR077288
		Baakal	BKAV	MN092352
		Koongol	KOOV	KP792669
		Wongal	WONV	MK896432
		Lednice	LEDV	MK514125
		M'Poko	MPOV	MK896536
		Umbre	UMBV	MK330169
		Little Sussex	LTLSV	OR077282
		Turlock	TURV	MK896444
		Gamboa	GAMV	KM272174
		Alajuela	ALJV	KM272186
		San Juan	SJV	MK896474
		Pueble Viejo	PVV	MK896486
		Calchaqui	CQIV	KM272183
		Bakau	BAKV	MK896635
		Ketapang	KETV	MK896548
		Nola	NOLAV	MK896512
		Tai orthobunyavirus	unavailable	OQ031273
		Telok Forest	TFV	MK896453
		Witwatersrand	WITV	KP792690
		Anopheles A	ANAV	KY793537
		Las Maloyas	LMV	MK896545
		Lukuni	LUKV	KP792672
		Anopheles B	ANBV	MK896644
		Boraceia	BORV	MK896611
		Triniti	TNTV	MG792213
		Tacaiuma	TCMV	MF497778
		Okola	OKOV	MK896509
		Tataguine	TATV	KP792678
		Tanga	TANV	MK896459
		Buffalo Creek	BUCV	KJ481929
		Trubanaman	TRUV	KR013235
		Gan Gan	GGV	KR013232
		Salt Ash	SASHV	KF234256
		Maprik	MPKV	KJ481926
		Mapputta	MAPV	KJ481923
Peribunyaviridae	*Pacuvirus*	Caimito	CAIV	MK330759
		Santarem	STMV	MK896471
		Tarpirape	TAPV	KM225260
		Chilibre	CHIV	MK330762
		Pacui	PACV	KM225254
		Rio Preto da Eva	RPEV	KM225257
Peribunyaviridae	*Gryffinivirus*	Asum	ASUMV	MK440651
		Hedwig	HEDV	OV054445
		Udune	UDNV	MW434620
Peribunyaviridae	*Kurdivirus*	Khurdun	KHURV	KF981633
Peribunyaviridae	*Herbevirus*	Herbert	HERV	JQ659256
		Kibale	KIBV	KF590577
		Tai	TAIV	KF590574
Peribunyaviridae	*Lakivirus*	Lakamha	LAKV	MN092355
Peribunyaviridae	*Shangavirus*	Shuangao insect virus 1	SgIV1	KM817679
Peribunyaviridae	*Lambavirus*	Largemouth bass	LBBV	MN119734
Flaviviridae	*Orthoflavivirus*	Turkish sheep encephalitis	TSEV	DQ235151
		Koutango	KOUV	EU082200
		Nakiwogo	NAKV	GQ165809
		Barkedji	BJV	KC496020
		Long Pine Key	LPKV	KY290249
		La Tina	LTNV	KY320649
		Marisma mosquito	MMV	KY347801
		Dengue 2	DENV-2	NC_001474
		West Nile	WNV	NC_001563
		Tick-borne encephalitis	TBEV	NC_001672

	Louping ill	LIV	NC_001809
	Rio Bravo	RBV	NC_003675
	Apoi	APOIV	NC_003676
	Powassan	POWV	NC_003687
	Tamana bat	TABV	NC_003996
	Montana myotis leukoencephalitis	MMLV	NC_004119
	Alkhurma	ALKV	NC_004355
	Yokose	YOKV	NC_005039
	Omsk hemorragic fever	OHFV	NC_005062
	Kamiti River	KRV	NC_005064
	Usutu	USUV	NC_006551
	Karshi	KSIV	NC_006947
	Aedes flavivirus	AEFV	NC_012932
	Chaoyang	CHAOV	NC_017086
	Mosquito flavivirus	MoFV	NC_021069
	Nhumirim	NHUV	NC_024017
	Ilomantsi	ILOV	NC_024805
	Lammi	LAMV	NC_024806
	Jutiapa	JUTV	NC_026620
	Cacipacore	CPCV	NC_026623
	Sokoluk	SOKV	NC_026624
	Spanish goat encephalitis	SGEV	NC_027709
	Potiskum	POTV	NC_029054
	Spondweni	SPOV	NC_029055
	Edge Hill	EHV	NC_030289
	Hanko	HaFV	NC_030401
	Bouboui	BOUV	NC_033693
	Palm Creek	PCFV	NC_033694
	Saboya	SABV	NC_033697
	Uganda S	UGSV	NC_033698
	Jugra	JUGV	NC_033699
	Meaban	MEAV	NC_033721
	Gadgets Gully	GGYV	NC_033723
	Kadam	KADV	NC_033724
	Saumarez Reef	SREV	NC_033726
	Phnom Penh bat	PPBV	NC_034007
	Yaounde	YAOV	NC_034018
	Kyasanur forest disease	KFDV	NC_039218
	Royal Farm	RFV	NC_039219
	Kampung Karu	KPKV	NC_040788
	Banzi	BANV	NC_043110
	Stratford	STRV	KM225263
	Torres	unavailable	KM225265
	Dengue 3	DENV-3	NC_001475
	Dengue 1	DENV-1	NC_001477
	Cell fusing agent	CFAV	NC_001564
	Yellow Fever	YFV	NC_002031
	Modoc	MODV	NC_003635
	Langat	LGTV	NC_003690
	Culex flavivirus	CXFV	NC_008604
	Zika	ZIKV	NC_012532
	Kedougou	KEDV	NC_012533
	Bagaza	BAGV	NC_012534
	Quang Binh	QBV	NC_012671
	Wesselsbron	WESSV	NC_012735
	Tembusu	TMUV	NC_015843
	Ntaya	NTAV	NC_018705
	Mercadeo	MECDV	NC_027819
	New Mapoon	NMV	NC_032088
	Nounane	NOUV	NC_033715
	T Ho	unavailable	NC_034151
	Ochlerotatus caspius flavivirus	OCFVPT	NC_034242
	Nanay	NANV	NC_040610
	Culex theileri flavivirus	CXthFV	NC_040682
	Iguape	IGUV	AY632538
	Alfuy	ALFV	AY898809
	Culex theileri	CXthFV	HE574574
	Bainyik	unavailable	KM225264

		Murray Valley encephalitis	MVEV	NC_000943
		Japanese encephalitis	JEV	NC_001437
		Dengue 4	DENV-4	NC_002640
		St Louis encephalitis	STLV	NC_007580
		Entebbe bat	ENTV	NC_008718
		Sepik	SEPV	NC_008719
		Bussuquara	BSQV	NC_009026
		Ilheus	ILHV	NC_009028
		Kokobera	KOKV	NC_009029
		Donggang	DONV	NC_016997
		Tyuleniy	TYUV	NC_023424
		Kama	KAMV	NC_023439
		Nienokoue	NiFV	NC_024299
		Paraiso Escondido	EPEV	NC_027999
		Rocio	ROCV	NC_040776
Togaviridae	*Alphavirus*	Middelburg	MIDV	EF536323
		Una	UNAV	HM147992
		Semliki forest	SFV	JF972635
		Ross River	RRV	GQ433354
		Getah	GETV	AY702913
		Sagiyama	SAGV	AB032553
		Bebaru	BEBV	HM147985
		Mayaro	MAYV	NC_003417
		Chikungunya	CHIKV	KP003813
		Onyong-nyong	ONNV	AF079456
		Igbo Ora	IGORV	AF079457
Phenuiviridae	*Uukuvirus*	Nile warbler	NIWV	HM566159
		Uukuniemi	UUKV	D10759
		Zaliv Terpeniya	TPV	HM566191
		Grand Arbaud	GAV	JF838327
		Murre	MURV	JF838330
		Precarious point	PPV	HM566181
		Rukutama	RUKV	KF892052
		Kabuto mountain	KAMV	LC153711
		Huangpi Tick 2	HpTV2	KM817668
		Silverwater	SILV	KM114257
		Kaisodi	KASDV	MG581739
		Toyo	TOYOV	LC618931
		Tongren Perib tick 2	TcTV2	UYL95521
		Dabieshan Tick	DbsTV; DBTV; DTV	KM817666
		Yongjia Tick 1	YONV	KM817704
		American dog tick	ADAV	KM048311
		Pacific coast tick phlebo	PACV	KU933936
		Tacheng Tick 2	TcTV2	KM817684
		Lihan Tick	LITV	KM817672
Nairoviridae	*Orthonairovirus*	Farallon	FARV	KU343154
		Hughes orthonairovirus	HUGV	AMT75407
		Raza	RAZAV	AMT75416
		Punta Salinas	PSV	AMT75410
		Zirqa	ZIRV	AMT75437
		Great Saltee	GRSV	AMT75404
		Caspiy	CSV	AKC89346
		Soldado	SOLV	AMT75425
		Estero Real	ERV	AXP33563
		Vinegar Hill	VINHV	AUD40046
		Dera Ghazi Khan	DGKV	KU343151
		Abu Hammad	AMV	AMT75371
		Abu Mina	AHV	AMT75374
		Saphire II	SAPV	AMT75422
Alpharhabdovirinae	*Vesiculovirus*	Malpais Spring	MSPV	KC412247
		Isfahan	ISFV	AJ810084
		Chandipura	CHNV	GU212856
		Piry	PIRYV	KU178986
		Perinet	PERV	HM566195
		Jurona	JURV	KM204996
		Mejal	MEJV	MW798173
		Radi	RADV	KM205024
		Yug Bogdanovac	YBV	JF911700

		vesicular stomatitis New Jersey	VSNJV	JX121109
		Carajás	CARV	KM205015
		vesicular stomatitis Alagoas	VSAV	EU373658
		Cocal	COCV	EU373657
		Maraba	MARV	HQ660076
		Morreton	MORV	KM205007
		vesicular stomatitis Indiana	VSIV	AF473864
		American bat vesiculovirus	ABV	JX569193
		Wufeng Myotis altarium vesiculovirus 1	WfMaIVV-1	OM030292
		Mediterranean bat	MBV	MW557336
		Jinghong bat	JhBV	MF279192
		Yinshui bat	YSBV	MN607594
Alpharhabdovirinae	*Sprivivirus*	pike fry rhabdo	PFRV	FJ872827
		spring viremia of carp	SVCV	U18101
Alpharhabdovirinae	*Siniperhavirus*	eelpout rhabdo	EPRV	KR612230
		Siniperca chuatsi rhabdovirus	SCRV	DQ399789
Alpharhabdovirinae	*Cetarhavirus*	dolphin rhabdovirus	DRV	KF958252
		harbour porpoise rhabdovirus	HPRV	MN103537
Alpharhabdovirinae	*Perhabdovirus*	eel virus European X	EVEX	FN557213
		lake trout rhabdovirus	STRV	MN963997
		perch rhabdovirus	PRV	JX679246
		Leman	LEMV	MN963996
Alpharhabdovirinae	*Uniorhavirus*	killamcar virus 1	KILLV1	OQ368743
Alpharhabdovirinae	*Scophrhavirus*	Scophthalmus maximus rhabdovirus	SMRV	HQ003891
		Wuhan redfin culter dimarhabdovirus	WhRCDRV	MG600013
Alpharhabdovirinae	*Ledantevirus*	Barur	BARV	KM204983
		Fukuoka	FUKV	KM205001
		Nishimuro	NISV	AB609604
		Nkolbisson	NKOV	KM205017
		Longquan Niviventer coninga ledantevirus 1	LNcLV1	MZ328293
		Tongren rhabd tick virus 2	TrRTV2	ON746534
		Yongjia tick virus 2	YTV2	KM817662
		Keuraliba	KEUV	KM205021
		Le Dantec	LDV	KM205006
		Vaprio	VAPV	MG021441
		Kern Canyon virus	KCV	KM204992
		Mount Elgon bat virus	MEBV	KM205026
		Taiyi bat	TYBV	MN607592
		Kanyawara	KYAV	KY385390
		Bughendera	BUGV	MT325641
		Wuhan louse fly virus 5	WLFV5	KM817654
		Oita	OITAV	KM204998
		Wenzhou Rhinolophus pusillus ledantevirus 1	WRpLV1	OM030289
		Kumasi rhabdovirus	KRV	KJ179955
		Fikirini	FKRV	KC676792
		Kolente	KOLEV	KC984953
Alpharhabdovirinae	*Alphathriprhavirus*	Hangzhou Frankliniella intonsa rhabdovirus 1	HzFiRV1	MZ209657
		Thrips tabaci associated dimarhabdovirus 1	TTaDRV1	MN714687
		Hubei lepidoptera virus 2	HbLV2	KX884415
		Pararge aegeria rhabdovirus	PAerRV	KR822826
Alpharhabdovirinae	*Sigmavirus*	Shayang fly virus 2	SyFV2	KM817635
		Wuhan fly virus 2	WhFV2	KM817646
		Yushu rhabdovirus	YsRV	MW826525
		Wuhan house fly virus 1	WhHFV1	KM817648
		Hubei diptera virus 10	HbDV10	KX884433
		Hubei diptera virus 9	HbDV9	KX884429
		Apis rhabdovirus 3	ApRV3	MZ822104
		Bactrocera tyroni rhabdovirus 1	BtyrRV1	MW208811
		Jopcycgri virus 1	JOPV1	ON324119

		Drosophila obscura sigmavirus	DObSV	GQ410979
		Drosophila immigrans sigmavirus	DImmSV	KX884434
		Drosophila sturtevanti sigmavirus	DStuSV	KR822816
		Drosophila affinis sigmavirus	DAffSV	KR822811
		Drosophila ananassae sigmavirus	DAnaSV	KR822812
		Ceratitis capitata sigmavirus	CCapSV	KR822825
		Hubei dimarhabdovirus 1	HbDRV1	KX884431
		Drosophila melanogaster sigmavirus	DMelSV	GQ375258
		Aksy-Durug Melophagus sigmavirus	ADMSV	OL420709
		Wuhan louse fly virus 9	WhLFV9	KM817656
		Wuhan louse fly virus 10	WhLFV10	KM817657
Alpharhabdovirinae	*Merhavirus*	Hattula rhabdovirus	HATTV	ON955142
		Inari rhabdovirus	INARV	ON955143
		Formosus	FORMV	BK059424
		Merida	MERDV	KU194360
		Culex tritaeniorhynchus rhabdovirus	CTRV	AB604791
Alpharhabdovirinae	*Ohlsrhavirus*	Lobeira	LOBV	MK780203
		Culex rhabdo-like virus Los Angeles	CRLVLA	MH188003
		Adumi	ADUMV	OQ077989
		Ohlsdorf	OHLDV	KY768856
		riverside	RISV	KU248085
		Culex pseudovishnui rhabdo-like	CpRLV	LC514057
		Tongilchon virus 1	TCHV	KU095840
		Culex rhabdo-like	CRLV	MF176333
		North Creek	NORCV	KF360973
Alpharhabdovirinae	*Caligrhavirus*	Caligus rogercresseyi rhabdovirus	CRogRV	KY203909
		Lepeophtheirus salmonis rhabdovirus 9	LSalRV9	KJ958535
		Lepeophtheirus salmonis rhabdovirus 127	LSalRV127	KJ958536
Alpharhabdovirinae	*Tupavirus*	Durham	DURV	FJ952155
		Klamath	KLAV	KM204999
		tupavirus SB8301	TUPVSB	OL774829
		tupaia rhabdovirus	TUPV	AY840978
		Wenzhou Myotis laniger tupavirus 1	WMlaTV1	OM030290
		bat tupavirus BS2	BtTVBS2	OQ709184
		Wufeng Rhinolophus pearsonii tupavirus 1	WRpeTV1	MZ328291
		bat tupavirus BS1	BtTVBS1	OQ709183
Alpharhabdovirinae	*Arurhavirus*	Aruac	ARUV	KM204987
		Xiburema	XIBV	KJ636781
		Inhangapi	INHV	KM204991
		Santa Barbara	SBAV	KM350503
Alpharhabdovirinae	*Curiovirus*	Itacaiunas	ITAV	KM204984
		Iriri	IRIRV	KM204995
		Curionopolis	CURV	KM204994
		Rochambeau	RBUV	KM205012
Alpharhabdovirinae	*Hapavirus*	Kamese	KAMV	KM204989
		Mossuril	MOSV	KM204993
		Flanders	FLAV	KM205002
		Hart Park	HPV	KM205011
		Mosqueiro	MQOV	KM205014
		Landjia	LJAV	KM205010
		Manitoba	MANV	KM205008
		Gray Lodge	GLOV	KM205022
		Porton	PORV	MW491751
		Marco	MCOV	KM205005
		La Joya	LJV	KM204986

		Bangoran	BGNV	MW491752
		Wongabel	WONV	EF612701
		Holmes Jungle	HOJV	KY421919
		Ord River	ORV	KM205025
		Parry Creek	PCV	KM204988
		Joinjakaka	JOIV	KM205016
		Ngaingan	NGAV	FJ715959
Alpharhabdovirinae	*Tibrovirus*	Beatrice Hill	BHV	KY073493
		Tibrogargan	TIBV	GQ294472
		Sweetwater Branch	SWBV	KM204997
		Coastal Plains	CPV	GQ294473
		Ekpoma virus 1	EKV1	KP324827
		Bas-Congo	BASV	JX297815
		Ekpoma virus 2	EKV2	KP324828
		Mundri	MUNV	OM320812
Alpharhabdovirinae	*Ephemerovirus*	Adelaide River	ARV	JN935380
		Obodhiang	OBOV	HM856902
		Kimberley	KIMV	JQ941664
		Huanggang rhabd tick virus 2	HgRTV2	ON746527
		bovine ephemeral fever	BEFV	AF234533
		Berrimah	BRMV	HM461974
		Hayes Yard	HYV	MH507506
		Puchong	PUCV	MH507505
		porcine ephemerovirus 1	PoEV1	OK086697
		porcine ephemerovirus 2	PoEV2	OK086698
		Yata	YATV	KM085030
		New Kent County	NKCV	MF615270
		Koolpinyah	KOOLV	KM085029
		kotonkan	KOTV	HM474855
Alpharhabdovirinae	*Sripuvirus*	Hainan black-spectacled toad rhabdovirus	HnBSTV	MG600016
		Charleville	CHVV	MH899109
		Almpiwar	ALMV	KJ399977
		Niakha	NIAV	KC585008
		Sripur	SRIV	KM205023
		Cuiaba	CUIV	MH89911
		Chaco	CHOV	KM205000
		Sena Madureira	SMV	KM205004
Alpharhabdovirinae	*Sunrhavirus*	Oak Vale	OVV	JF705876
		Dillard's Draw	DDRV	MG251664
		Kwatta	KWATV	KM204985
		Harrison Dam	HARDV	KJ432573
		Walkabout Creek	WACV	KJ432572
		Boteke	BOTV	MW491753
		Sunguru	SUNV	KF395226
		Bimbo	BBOV	MW491756
		Kolongo	KOLV	MW491757
		Ouango	OUAV	MW491758
		Sandjimba	SJAV	MW491754
		Nasoule	NASV	MW491755
		Burg el Arab	BEAV	MW491759
		Garba	GARV	KM204982
		Matariya	MTYV	MW491760
Alpharhabdovirinae	*Alphanemrhavirus*	Xingshan nematode virus 4	XsNV4	KX884459
		Sodak rhabdovirus 1	SDRV1	MT875151
		Rattus tanezumi rhabdovirus 1	RtaRV1	MT085340
		Xinzhou nematode virus 4	XzNV4	KX884462
		Taishun tick	TsTV	KM817643
		Zhangjiakou rhabd tick virus 1	ZjRTV1	ON746531
		Kioloa tick	HbanRV	OK128265
		Huangpi tick virus 3	HpTV3	KM817630
		Norway mononegavirus 1	NWMNV1	MF141072
		Yanbian rhabd tick virus 1	YbRTV1	ON746520
		Nanning rhabd tick virus 1	NnRTV1	ON746519
		Tahe rhabdovirus 1	ThRV1	ON408166
		Huanggang rhabd tick virus 1	HgRTV1	ON746518
		Dermacentor reticulatus rhabdovirus 1	DretRV1	ON684361

		Yushu rhabd tick virus 2	YsRTV2	ON746530
		Wuhan tick virus 1	WhTV1	KM817660
		Blanchseco	BCOV	MN025503
		Bole tick virus 2	BlTV2_L	KM817629
		Hubei tick rhabdovirus 1	HbTRV1	MW721934
		Guyuan rhabd tick virus 1	GyRTV1	ON746521
Alpharhabdovirinae	*Sawgrhavirus*	Connecticut	CNTV	KM205020
		lone star tick rhabdovirus	LITRV	KJ396935
		Sawgrass	SAWV	KM205013
		New Minto	NMV	KM205009
Alpharhabdovirinae	*Mousrhavirus*	Moussa	MOUV	FJ985748
Alpharhabdovirinae	*Barhavirus*	Muir Springs	MSV	KM204990
		Bahia Grande	BGV	KM205018
Alpharhabdovirinae	*Zarhavirus*	Zahedan rhabdovirus	ZAHV	KJ830812
Alpharhabdovirinae	*Lostrhavirus*	lone star tick rhabdovirus	LSTRV	KU127239
		Xinjiang tick rhabdovirus	XjTRV	MH688524
		Alxa tick rhabdovirus	ATRV	OP313011
Alpharhabdovirinae	*Amplylivirus*	frog lyssa-like virus 1	FLLV1	MK473367
		Boana pugnax lyssa-like virus 1	BpugLLV1	MZ682616
Alpharhabdovirinae	*Replylivirus*	anole lyssa-like virus 1	ALLV1	BR001666
Alpharhabdovirinae	*Lyssavirus*	Ikoma lyssavirus	IKOV	JX193798
		Lleida bat lyssavirus	LLEBV	KY006983
		West Caucasian bat	WCBV	EF614258
		Shimoni bat	SHIBV	GU170201
		Lagos bat	LBV	EU293108
		Mokola	MOKV	Y09762
		European bat lyssavirus 1	EBLV1	EF157976
		Irkut	IRKV	EF614260
		Taiwan bat lyssavirus	TWBLV	MF472710
		Duvenhage	DUVV	EU293119
		rabies	RABV	M13215
		Gannoruwa bat lyssavirus	GBLV	KU244266
		Australian bat lyssavirus	ABLV	AF081020
		European bat lyssavirus 2	EBLV2	EF157977
		Bokeloh bat lyssavirus	BBLV	JF311903
		Aravan	ARAV	EF614259
		Khujand	KHUV	EF614261
		Kotalahti bat lyssavirus	KBLV	LR994545
Alpharhabdovirinae	*Almendravirus*	Arboretum	ABTV	KC994644
		Shanxi arboretum	SxABV	MW890015
		Puerto Almendras	PTAMV	KF534749
		Coot Bay	CBV	KX228196
		Menghai rhabdovirus	MRV	KX785335
		Balsa	BALV	KX228198
		Rio Chico	RCHV	KX228197
		Xiangshan rhabdo-like virus 1	XsRLV1	OK491499
Alpharhabdovirinae	*Alphaplatrhavirus*	Schistorhabdovirus 1	SchRV	BK059758
		Sphaeridiorhabdovirus 1	SphRV1	BK059662
		Microrhabdovirus 1	MicRV	BK059723
		Metorhabdovirus 2	MetRV2	BK059676
		fox fecal rhabdovirus	FFRV	KF823814
		Tritaenorhabdovirus 1	TriRV1	BK059681
Deltarhabdovirinae	*Betaricinrhavirus*	Chimay rhabdovirus	CRV	MF975531
		Mudanjiang rhabd tick virus 1	MjRTV1	ON746525
		blacklegged tick rhabdovirus 1	BLTRV1	MF360790
		Tongren rhabd tick virus 1	TrRTV1	ON746523
		Yanbian rhabd tick virus 3	YbRTV3	ON746524
Deltarhabdovirinae	*Gammahymrhavirus*	Diachasminorpha longicaudata rhabdovirus	DlonRV	KP735609
		Apis rhabdovirus 4	ApRV4	MZ822105
		Apis rhabdovirus 5	ApRV5	MZ822106
Deltarhabdovirinae	*Alphacrustrhavirus*	Wenling crustacean virus 10	WlCV10	KX884450
		Wenling crustacean virus 11	WlCV11	KX884456
		Shayang fly virus 3	SyFV3	KM817636
Deltarhabdovirinae	*Alphadrosrhavirus*	Wuhan fly virus 2	WHFV2	KM817649
Deltarhabdovirinae	*Betahymrhavirus*	hymenopteran rhabdo-related virus 23	HyRRV23	MW314717

		hymenopteran rhabdo-related virus 24	HyRRV24	MW039260
		Xiangshan rhabdo-like virus 4	XsRLV4	OK491502
Deltarhabdovirinae	*Stangrhavirus*	Guadeloupe Culex rhabdovirus	GCRV	MN013386
		Wuhan mosquito virus 9	WnMV9	OL700091
		Elisy	ELSYV	MW434768
		Stang	STNGV	MW434775
Deltarhabdovirinae	*Betapaprhavirus*	Spodoptera frugiperda rhabdovirus	SfruRV	KF947078
		Lepidopteran rhabdo-related virus 34	LeRRV34	MT153466
Deltarhabdovirinae	*Primrhavirus*	Primus	PRIMV	MN567480
		San Gabriel mononegavirus	SGMNV	BK059423
		Atrato rhabdo-like virus 3	AtRLV3	MN661034
Deltarhabdovirinae	*Alphahymrhavirus*	Lasius neglectus virus 2	LnegV2	MH477288
		Hymenopteran rhabdo-related virus 38	HyRRV38	MT153454
		Xiangshan rhabdo-like virus 3	XsRLV3	OK491501
		Lariophagus distinguendus negative strand RNA virus 1	LdisNSRV1	MW864604
		Hymenopteran rhabdo-related virus 46	HyRRV46	MW314718
		Hymenopteran rhabdo-related virus 109	HyRRV109	MT153372
Deltarhabdovirinae	*Betanemrhavirus*	Hubei rhabdo-like virus 9	HbRLV9	KX884448
		Shayang ascaridia galli virus 2	SyAGV2	KX884414
Deltarhabdovirinae	*Gammaricinrhavirus*	Fuyun tick rhabdovirus	FyTRV	OP313012
		Tacheng tick virus 7	TcTV7	KM817642
Betarhabdovirinae	*Varicosavirus*	Brassica rapa virus 1	BrRV1	BK014310
		Raphanus virus 1	RapV1	BK061799
		Erysimum virus 1	EryV1	BK061766
		Aponogeton virus 1	ApoV1	BK061739
		Centaurea virus 1	CenV1	BK061757
		Silene virus 1	SilV1	BK061807
		Melilotus virus 1	MelV1	BK061784
		Red clover associated varicosavirus	RCaVV	MF918568
		Triticum virus 1	TriV1	BK061821
		Caladenia virus 1	CalV1	BK061755
		Artemisia virus 1	ArtV1	BK061741
		Tanacetum virus 1	TanV1	BK061815
		Leucanthemum virus 1	LeuV1	BK061774
		Ribes virus 1	RibV-1	BK061801
		Ranunculus virus 1	RanV1	BK061797
		Vicetoxicum virus 1	VinV1	BK061823
		Morning glory varicosavirus	MGVV	MW922438
		Apera virus 1	ApeV1	BK061737
		Melampyrum roseum virus 1	MelRoV1	BK014314
		Guizotia virus 1	GuiV1	BK061770
		Vitis varicosavirus	VVV	LC604719
		Zostera-associated varicosavirus 1	ZaVV1	BK014484
		Aconitum virus 1	AcoV1	BK061734
		Arceuthobium virus 8	ArcV8	BK061732
		Didymochlaena virus 1	DidV1	BK061764
		Tree fern varicosa-like	TfVV	OW528630
		Treubia virus 1	TreV1	BK061819
		Frullania virus 1	FruV1	BK061768
		Morning glory varicosavirus	MgVV	OW528612
		Allium angulosum virus 1	AanV1	BK059208
		Luffa virus 1	LufV1	BK061780
		Cucumis virus 1	CucmV1	BK061761
Betarhabdovirinae	*Alphacytorhabdovirus*	Strawberry virus 1	StrV1	BK061813
Betarhabdovirinae	*Varicosavirus*	Zea virus 1	ZeaV1	BK061825
		Pennisetum virus 1	PenV1	BK061790

		Holcus virus 1	HolV1	BK061772
		Alopecurus myosuroides varicosavirus 1	AMVV1	LN713933
		Lolium perenne virus 1	LoPV1	BK014312
		Brassica virus 2	BrV2	BK061747
		Primula virus 1	PriV1	BK061795
		Asclepias syriaca virus 3	AscSyV3	BK061743
		Lettuce big vein-associated	LBVaV	AB075039
		Xinjiang varicosavirus	XVV	MW897032
Betarhabdovirinae	*Betagymnorhavirus*	Torreya virus 1	TorV-1	BK061818
Betarhabdovirinae	*Alphagymnorhavirus*	Sciadopitys virus 1	SciV1	BK061803
		Taxus virus 1	TaxV-1	BK061817
		Amentotaxus virus 1	AmeV1	BK061736
		Cupressus virus 1	CupV1	BK061763
		Picea virus 1	PicV-1	BK061792
		Abies virus 1	AbiV1	BK061731
		Pinus banksiana virus 1	PiBanV1	BK061793
		Pinus flexilis virus 1	PiFleV1	BK014316
		Pinis yunannensis virus 1	PiYunV1	BK061794
Betarhabdovirinae	*Betanucleorhabdovirus*	Zhuye pepper nucleorhabdovirus	ZPNRV	MH323437
		Birds-foot trefoil-associated	BFTV	BK010826
		Sowthistle yellow vein	SYVV	MT185675
		Cnidium virus 1	CnV1	MZ983390
		Cardamom vein clearing	CdVCV	MN273311
		Blackcurrant-associated rhabdovirus	BCaRV	MF543022
		Tomato betanucleorhabdovirus 1	TBRV1	OL472119
		Tomato betanucleorhabdovirus 2	TBRV2	OL472114
		Plectranthus aromaticus virus 1	PleArV1	BK014300
		Datura yellow vein	DYVV	KM823531
		Bacopa monnieri virus 2	BmV2	BK014480
		Sonchus yellow net virus	SYNV	L32603
		Taraxacum betanucleorhabdvirus 1	TarBRV1	OL472118
		Rhododendon delavayi virus 1	RhoDeV1	BK014301
		Picris betanucleorhabdvirus 1	PBRV1	OL472117
		Apple rootstock virus A	ApRVA	MH778545
		Asclepias syriaca virus 2	AscSyV2	BK014299
		Alfalfa-associated nucleorhabdovirus	AaNV	MG948563
Betarhabdovirinae	*Dichorhavirus*	Orchid fleck	OFV	AB244418
		Citrus leprosis virus N	CiLVN	KX982179
		Citrus bright spot	CiBSV	MZ773938
		Citrus chlorotic spot	CiCSV	KY700686
		Coffee ringspot	CoRSV	KF812526
		Clerodendrum chlorotic spot	ClCSV	MG938507
Betarhabdovirinae	*Gammanucleorhabdovirus*	Maize fine streak	MFSV	AY618417
		Cereal chlorotic mottle	CCMoV	MW731536
Betarhabdovirinae	*Alphanucleorhabdovirus*	Taro vein chlorosis	TaVCV	AY674964
		Morogoro maize-associated	MMaV	MK063878
		Maize mosaic	MMV	AY618418
		Maize Iranian mosaic	MIMV	MF102281
		Agave tequilana virus 1	ATV1	BK014297
		Peach virus 1	PeV1	MN520414
		Rice yellow stunt	RYSV	AB011257
		Wheat yellow striate	WYSV	MG604920
		Artemisia capillaris nucleorhabdovirus 1	ArtCaNV1	OM372677
		Xinjiang alphanucleorhabdovirus	XARV	MW897039
		Constricta yellow dwarf	CYDV	KY549567
		Joa yellow blotch associated	JYBaV	MW014292
		Potato yellow dwarf	PYDV	GU734660
		Physostegia chlorotic mottle	PhCMoV	KX636164
		Eggplant mottled dwarf	EMDV	KJ082087

		Tomato alphanucleorhabdovirus 1	TARV1	OL472126
Betarhabdovirinae	*Deltanucleorhabdovirus*	Medicago sativa virus 1	MSV1	ON246246
		Strawberry virus 3	StrV3	MW503935
Betarhabdovirinae	*Betacytorhabdovirus*	Barley yellow striate mosaic	BYSMV	KM213865
		Maize yellow striate	MYSV	KY884303
		Northern cereal mosaic	NCMV	AB030277
		Maize-associated cytorhabdovirus	MaCV	KY965147
		Colocasia bobone disease-associated	CBDaV	KT381973
		Rose virus R	RVR	MT952336
		Tagetes erecta virus 1	TaEV1	BK014308
		Rudbeckia virus 1	RudV1	ON185810
		Paper mulberry mosaic associated	PMuMaV	MN872813
		Anthurium amnicola virus 1	AntAmV1	BK014302
		Rice stripe mosaic	RSMV	KX525586
		Papaya virus E	PpVE	MH282832
		Tilia cytorhabdovirus 1	TiCRV1	OX411436
		Yerba mate chlorosis-associated	YmCaV	KY366322
		Soybean blotchy mosaic	SbBMV	OM681518
		Cucurbit cytorhabdovirus 1	CuCV1	MT381995
		Bemisia tabaci-associated virus 1	BeTaV1	BK014303
		Yerba mate virus A	YmVA	MN781667
		Rose-associated cytorhabdovirus	RaCV	ON762421
Betarhabdovirinae	*Gammacytorhabdovirus*	Gymnadenia densiflora virus 1	GymDenV1	BK014305
		Trachyspermum ammi virus 1	TrAV1	BK014309
Betarhabdovirinae	*Alphacytorhabdovirus*	Asclepias syriaca virus 1	AscSyV1	BK014298
		Daphne virus 1	DV1	OP180101
		Bacopa monnieri virus 1	BmV1	BK014479
		Wuhan insect virus 4	WhIV4	KM817650
		Actinidia virus D	AcVD	MW550041
		Taraxacum cytorhabdovirus 1	TCRV1	OL472125
		Lettuce yellow mottle	LYMoV	EF687738
		Trifolium pratense virus B	TpVB	MH982249
		Lettuce necrotic yellows virus	LNYV	AJ867584
		Blackcurrant rhabdovirus 2	BCRV2	OP352885
		Strawberry virus 2	StrV2	MW480851
		Cabbage cytorhabdovirus 1	CCyV	KY810772
		Trichosanthes associated rhabdovirus 1	TrARV1	BK011194
		Strawberry-associated virus 1	SaV1	OM523356
		Wuhan insect virus 5	WhIV5	KM817651
		Persimmon virus A	PeVA	AB735628
		Alfalfa dwarf	ADV	KP205452
		Raspberry vein chlorosis	RVCV	MK240091
		Strawberry crinkle	SCV	MH129615
		Hyptis latent	HpLV	ON073823
		Chrysanthemum yellow dwarf associated	CYDaV	MW039593
		Pastinaca cytorhabdovirus 1	PaCRV1	OL472112
		Tomato yellow mottle-associated	TYMaV	KY075646
		Patchouly chlorosis-associated cytorhabdovirus	PCaCV	ON409991
		Strawberry virus 1	StrV1	MK211271
		Kenyan potato cytorhabdovirus	KePCyV	MN689395
		Nymphaea alba virus 1	NymAV1	BK014307
		Wuhan insect virus 6	WhIV6	KM817652
		Trifolium pratense virus A	TpVA	MH982250
		Glehnia littoralis virus 1	GlLV1	BK014304
Gammarhabdovirinae	*Margarhavirus*	Chemarfal virus 1	CHMFV1	OQ368744
Gammarhabdovirinae	*Novirhabdovirus*	Hirame rhabdovirus	HIRRV	AF104985

	Infectious hematopoietic necrosis	IHNV	L40883
	Viral hemorrhagic septicemia	VHSV	Y18263
	Snakehead rhabdovirus	SHRV	AF147498

Supplementary Table 6. Reassortment analysis using RDP5 (81) for *Orthobunyavirus* strains. The table displays p-values from multiple detection methods, including RDP, GENECONV, Bootscan, Maxchi, Chimaera, SiSscan, PhylPro, LARD, and 3Seq.

Detection methods	Brus Laguna virus reassortment p-values*	Ossa virus reassortment p-values*
RDP	2.19×10^{-23}	5.63×10^{-34}
GENECONV	NS	8.39×10^{-13}
Bootscan	1.88×10^{-8}	7.71×10^{-39}
Maxchi	3.13×10^{-280}	4.77×10^{-33}
Chimaera	1.54×10^{-18}	3.50×10^{-27}
SiSscan	3.36×10^{-56}	8.84×10^{-54}
PhylPro, LARD, 3Seq	NS	2.54×10^{-15}

Supplementary Table 7. Genome sequences used in the *Orthobunyavirus* family reassortment analyses (81).

Virus name	GenBank ID Segment L	GenBank ID Segment M	GenBank ID Segment S
Baakal	MN092352	MN092353	MN092354
Minatitlan	MK896527	MK896526	MK896525
Mirim	KY013487	KY013488	KY013489
Bertioga	MK896614	MK896613	MK896612
Guaratuba	MK896570	MK896569	MK896568
Itimirim	MK896557	MK896556	MK896555
Tacaiuma	MF497778	MF497776	MF497775
Anopheles B	MK896644	MK896643	MK896642
Boraceia	MK896611	MK896610	MK896609
Anopheles A	KY793537	KY793538	KY793539
Lukuni	KP792672	KP792671	KP792670
Las Maloyas	MK896545	MK896544	MK896543
Tataguine	KP792678	KP792677	KP792676
Triniti	MG792213	MG792214	MG792215
Okola	MK896509	MK896508	MK896507
Tanga	MK896459	MK896458	MK896457
Pueblo Viejo	MK896486	MK896485	MK896484
San Juan	MK896474	MK896473	MK896472
Alajuela	KM272186	KM272187	KM272188
Gamboa	KM272174	KM272175	KM272176
Tete	KP792681	KP792680	KP792679
Batama	MK896626	MK896625	MK896624
Lichuan	MT198373	MT198372	MT198371
Bahig	KP792654	KP792653	KP792652
Matruh	KP792693	KP792692	KP792691
I612045	HM627179	HM627181	HM627180

Orthobunyavirus reassortment analysis of 26 representative genomes, including two new genomes (Ossa and Brus Laguna) generated in this study,* ***Table S7**.*

Supplementary Table 8. Zoonotic rank analysis. Predicted probabilities of human infection, zoonotic potential categories, and relative priority ranks for all viruses in the manuscript, derived from the combined genome feature–based model.

Virus	Calibrated score mean	Calibrated score lower	Calibrated score upper	Bagged prediction	Priority category
Acado	2.942	1.307	5.393	TRUE	High
Andasibe	3.251	1.445	5.782	TRUE	High
Arboledas	4.253	2.445	7.121	TRUE	High
Arkonam	1.981	1.134	3.289	FALSE	Medium
Bauline	4.695	1.775	7.877	TRUE	High
Bobia	2.732	1.444	4.339	FALSE	Medium
Botambi	3.949	2.060	6.776	TRUE	High

Brus Laguna	3.075	1.755	5.639	TRUE	High
Cape Wrath	3.612	1.777	6.618	TRUE	High
Cowbone Ridge	2.799	1.158	5.122	FALSE	Medium
Csiro Village	3.539	1.614	5.750	TRUE	High
D'Aguilar	4.157	1.684	7.195	TRUE	High
Gomoka	3.585	1.777	6.618	TRUE	High
Huacho	2.953	1.456	5.676	TRUE	High
Inini	4.202	2.334	6.978	TRUE	High
Jacareacanga	2.959	1.602	5.104	TRUE	High
Kasba	2.450	1.110	4.395	FALSE	Medium
Kindia	3.431	1.342	6.253	TRUE	High
Kununurra	1.977	1.148	3.326	FALSE	Medium
Lake Clarendon	2.255	1.223	3.727	FALSE	Medium
Lanjan	6.118	3.654	8.595	TRUE	Very high
Llano Seco	2.899	1.542	5.017	FALSE	Medium
Marrakai	3.523	1.402	6.067	TRUE	High
Matariya	2.273	1.295	4.038	FALSE	Medium
Minnal	2.261	1.265	3.831	FALSE	Medium
Mitchell River	3.341	1.772	5.580	TRUE	High
Mono Lake	1.993	886	3.366	FALSE	Medium
Monte Dourado	3.569	1.874	5.979	TRUE	High
Mykines	3.877	1.504	6.779	TRUE	High
Ndelle	1.622	853	2.905	FALSE	Low
Nugget	3.024	1.351	5.099	TRUE	High
Olifantsvlei	2.373	1.133	3.702	FALSE	Medium
Ossa	3.426	1.626	5.612	TRUE	High
Palestina	3.297	1.599	5.266	TRUE	High
Paroo River	1.922	1.096	3.019	FALSE	Medium
Porton's	1.590	697	2.743	FALSE	Low
Seletar	2.277	1.211	3.709	FALSE	Medium
Sixgun City	2.057	925	3.552	FALSE	Medium
Timbo	1.772	972	2.915	FALSE	Low
Tindholmur	4.707	1.575	7.935	TRUE	High
Tsuruse	2.552	1.178	4.415	FALSE	Medium
Vellore	3.385	1.394	5.696	TRUE	High
Virgin River	4.197	2.085	6.733	TRUE	High
Xingu	3.730	1.951	6.463	TRUE	High
Yaquina Head	4.116	1.480	7.087	TRUE	High
Zingilamo	4.918	2.549	7.591	TRUE	High

References

1. Lefkowitz EJ, Dempsey DM, Hendrickson RC, Orton RJ, Siddell SG, Smith DB. 2018. Virus taxonomy: the database of the International Committee on Taxonomy of Viruses (ICTV). Nucleic Acids Res 46:D708-D717.
2. Matthijnssens J, Attoui H, Banyai K, Brussaard CPD, Danthi P, Del Vas M, Dermody TS, Duncan R, Fang Q, Johne R, Mertens PPC, Mohd Jaafar F, Patton JT, Sasaya T, Suzuki N, Wei T. 2022. ICTV Virus Taxonomy Profile: Spinareoviridae 2022. J Gen Virol 103.
3. Kuhn JH, Alkhovsky SV, Avsic-Zupanc T, Bergeron E, Burt F, Ergunay K, Garrison AR, Marklewitz M, Mirazimi A, Papa A, Paweska JT, Spengler JR, Palacios G. 2024. ICTV Virus Taxonomy Profile: Nairoviridae 2024. J Gen Virol 105.
4. Sasaya T, Palacios G, Briese T, Di Serio F, Groschup MH, Neriya Y, Song JW, Tomitaka Y. 2023. ICTV Virus Taxonomy Profile: Phenuiviridae 2023. J Gen Virol 104.
5. Walker PJ, Freitas-Astua J, Bejerman N, Blasdell KR, Breyta R, Dietzgen RG, Fooks AR, Kondo H, Kurath G, Kuzmin IV, Ramos-Gonzalez PL, Shi M, Stone DM, Tesh RB, Tordo N, Vasilakis N, Whitfield AE, Ictv Report C. 2022. ICTV Virus Taxonomy Profile: Rhabdoviridae 2022. J Gen Virol 103.
6. Simmonds P, Becher P, Bukh J, Gould EA, Meyers G, Monath T, Muerhoff S, Pletnev A, Rico-Hesse R, Smith DB, Stapleton JT, Ictv Report C. 2017. ICTV Virus Taxonomy Profile: Flaviviridae. J Gen Virol 98:2-3.

7. Chen R, Mukhopadhyay S, Merits A, Bolling B, Nasar F, Coffey LL, Powers A, Weaver SC, Ictv Report C. 2018. ICTV Virus Taxonomy Profile: Togaviridae. J Gen Virol 99:761-762.
8. Schmidt JRW, M.C.; Lule, M.; Mivule, A.; Mujomba, E. 1966. Viruses isolated from mosquitoes collected in southern Sudan and western Ethiopia. East African Virus Research Institute, Entebbe, Uganda.
9. Borden EC, Shope RE, Murphy FA. 1971. Physicochemical and morphological relationships of some arthropod-borne viruses to bluetongue virus--a new taxonomic group. Physiocochemical and serological studies. J Gen Virol 13:261-71.
10. Murphy FA, Borden EC, Shope RE, Harrison A. 1971. Physicochemical and morphological relationships of some arthropod-borne viruses to bluetongue virus--a new taxonomic group. Electron microscopic studies. J Gen Virol 13:273-88.
11. Clerc YA, H.; Andrianarivelo, M.; Rabarisoa, R. 1983 (1984). Archives de l'Institut Pasteur de Madagascar 51:135–138.
12. Tesh RB, Boshell J, Young DG, Morales A, Corredor A, Modi GB, Ferro de Carrasquilla C, de Rodriquez C, Gaitan MO. 1986. Biology of Arboledas virus, a new phlebotomus fever serogroup virus (Bunyaviridae: Phlebovirus) isolated from sand flies in Colombia. Am J Trop Med Hyg 35:1310-6.
13. Dandawate CN, Shope RE. 1975. Studies on physicochemical and biological properties of two ungrouped arboviruses: Minnal and Arkonam. Indian J Med Res 63:1180-7.
14. Main AJ, Downs WG, Shope RE, Wallis RC. 1973. Great Island and Bauline: two new Kemerovo group orbiviruses from Ixodes uriae in eastern Canada. J Med Entomol 10:229-35.
15. Main AJ, Downs WG, Shope RE, Wallis RC. 1976. Avian arboviruses of the Witless Bay seabird Sanctuary, Newfoundland, Canada. J Wildl Dis 12:182-94.
16. Main AJ, Shope RE, Wallis RC. 1976. Cape wrath: a new Kemerovo group orbivirus from Ixodes uriae (Acari: Ixodidae) in Scotland. J Med Entomol 13:304-8.
17. Digoutte JP. 1969. Annual Report, Institut Pasteur de Bangui. Bangui IPd,
18. Bangui IPd. 1968. Rapport Annuel de l'Institut Pasteur de Bangui. Bangui IPd, Bangui.
19. Robin Y. 1973. Annual Report of the Institut Pasteur de Dakar. Dakar IPd, Dakar.
20. Calisher CH, Lazuick JS, Sudia WD. 1988. Brus Laguna virus, a Gamboa bunyavirus from Aedeomyia squamipennis collected in Honduras. Am J Trop Med Hyg 39:406-8.
21. Calisher CH, Davie J, Coleman PH, Lord RD, Work TH. 1969. Cowbone Ridge virus, a new group B arbovirus from South Florida. Am J Epidemiol 89:211-6.
22. Varelas-Wesley I, Calisher CH. 1982. Antigenic relationships of flaviviruses with undetermined arthropod-borne status. Am J Trop Med Hyg 31:1273-84.
23. Knudson DL, Tesh RB, Main AJ, St George TD, Digoutte JP. 1984. Characterization of the Palyam serogroup viruses (Reoviridae: Orbivirus). Intervirology 22:41-9.
24. Cybinski DH, St George TD. 1982. Preliminary characterization of D'Aguilar virus and three Palyam group viruses new to Australia. Aust J Biol Sci 35:343-51.
25. Doherty RL, Carley JG, Standfast HA, Dyce AL, Snowdon WA. 1972. Virus strains isolated from arthropods during an epizootic of bovine ephemeral fever in Queensland. Aust Vet J 48:81-6.
26. Westaway EG. 1966. Assessment and application of a cell line from pig kidney for plaque assay and neutralization tests with twelve group B arboviruses. Am J Epidemiol 84:439-56.
27. Carley JG, Standfast HA, Kay BH. 1973. Multiplication of viruses isolated from arthopods and vertebrates in Australia in experimentally infected mosquitoes. J Med Entomol 10:244-9.
28. Schnagl RD, Holmes IH. 1971. A study of Australian arboviruses resembling bluetongue virus. Aust J Biol Sci 24:1151-62.
29. St George TD, Dimmock CK. 1976. The isolation of D'Aguilar virus from a cow. Aust Vet J 52:598.

30. Zeller HG, Karabatsos N, Calisher CH, Digoutte JP, Cropp CB, Murphy FA, Shope RE. 1989. Electron microscopic and antigenic studies of uncharacterized viruses. III. Evidence suggesting the placement of viruses in the family Reoviridae. Arch Virol 109:253-61.
31. Bangui IPd. 1970. Rapport Annuel de l'Institut Pasteur de Bangui. Institut Pasteur de Bangui, Bangui, Central African Republic.
32. Johnson HNaC, J. 1972. Transcontinental Connections of Migratory Birds and their Role in the Distribution of Arboviruses. *In* Cherepanov AI, et al. (ed), Transcontinental Connections of Migratory Birds and their Role in the Distribution of Arboviruses. Publishing House "NAUKA", Siberian Branch, Novosibirsk.
33. Digoutte JP. 1973. Rapport Annuel de l'Institut Pasteur de la Guyane Française. Institut Pasteur de la Guyane Française, Cayenne.
34. Digoutte JP. 1975. Rapport Annuel de l'Institut Pasteur de la Guyane Française. Institut Pasteur de la Guyane Française, Cayenne.
35. Dandawate CN, Rajagopalan PK, Pavri KM, Work TH. 1969. Virus isolations from mosquitoes collected in North Arcot district, Madras state, and Chittoor district, Andhra Pradesh between November 1955 and October 1957. Indian J Med Res 57:1420-6.
36. Dandawate CN. 1974. Antigenic relationship among Palyam, Kasba and Vellore viruses--a new serogroup of arboviruses. Indian J Med Res 62:326-31.
37. Dakar IPd. 1985. Institut Pasteur de Dakar Annual Report.
38. Dakar IPd. 1984. Institut Pasteur de Dakar Annual Report.
39. Liehne CG, Leivers S, Stanley NF, Alpers MP, Paul S, Liehne PF, Chan KH. 1976. Ord River arboviruses--isolations from mosquitoes. Aust J Exp Biol Med Sci 54:499-504.
40. St George TD, Cybinski DH, Main AJ, McKilligan N, Kemp DH. 1984. Isolation of a new arbovirus from the tick Argas robertsi from a cattle egret (Bubulcus ibis coromandus) colony in Australia. Aust J Biol Sci 37:85-9.
41. Tan DS, Smith CE, McMahon DA, Bowen ET. 1967. Lanjan virus, a new agent isolated from Dermacentor auratus in Malaya. Nature 214:1154-5.
42. Pavri KM, Casals J. 1966. Kaisodi virus, a new agent isolated from Haemaphysalis spinigera in Mysore state, South India. Am J Trop Med Hyg 15:961-3.
43. Stim TB. 1969. Arbovirus plaquing in two simian kidney cell lines. Journal of General Virology 5:329–338.
44. Gubler DJ, Rosen L. 1976. A simple technique for demonstrating transmission of dengue virus by mosquitoes without the use of vertebrate hosts. Am J Trop Med Hyg 25:146-50.
45. Tesh RB, Peleg J, Samina I, Margalit J, Bodkin DK, Shope RE, Knudson D. 1986. Biological and antigenic characterization of Netivot virus, an unusual new Orbivirus recovered from mosquitoes in Israel. Am J Trop Med Hyg 35:418-28.
46. Anonymous. 1980. Laboratory safety for arboviruses and certain other viruses of vertebrates. The Subcommittee on Arbovirus Laboratory Safety of the American Committee on Arthropod-Borne Viruses. Am J Trop Med Hyg 29:1359-81.
47. Doherty RL, Carley JG, Standfast HA, Dyce AL, Kay BH, Snowdon WA. 1973. Isolation of arboviruses from mosquitoes, biting midges, sandflies and vertebrates collected in Queensland, 1969 and 1970. Trans R Soc Trop Med Hyg 67:536-43.
48. Gonzalez HA, Knudson DL. 1988. Intra- and inter-serogroup genetic relatedness of orbiviruses. I. Blot hybridization of viruses of Australian serogroups. J Gen Virol 69 (Pt 1):125-34.
49. Travassos da Rosa AP, Tesh RB, Pinheiro FP, Travassos da Rosa JF, Peralta PH, Knudson DL. 1984. Characterization of the Changuinola serogroup viruses (Reoviridae: Orbivirus). Intervirology 21:38-49.
50. Spence RP, Harrap KA, Nuttall PA. 1985. The isolation of Kemerovo group orbiviruses and Uukuniemi group viruses of the family bunyaviridae from Ixodes uriae ticks from the Isle of May, Scotland. Acta Virol 29:129-36.
51. Moss SRP, Æ.; Nuttall, P. A. 1986. Tick-borne viruses in Icelandic seabird colonies. Acta Naturalia Islandica 32:1-19.

52. Dakar IP. 1975. Institut Pasteur Dakar Annual Report. Dakar, Senegal.
53. Doherty RL, Carley JG, Murray MD, Main AJ, Jr., Kay BH, Domrow R. 1975. Isolation of arboviruses (Kemerovo group, Sakhalin group) from Ixodes uriae collected at Macquarie Island, Southern ocean. Am J Trop Med Hyg 24:521-6.
54. Murphy FA, Harrison AK, Whitfield SG. 1973. Bunyaviridae: morphologic and morphogenetic similarities of Bunyamwera serologic supergroup viruses and several other arthropod-borne viruses. Intervirology 1:297-316.
55. Porterfield JS, Casals J, Chumakov MP, Gaidamovich SY, Hannoun C, Holmes IH, Horzinek MC, Mussgay M, Russell PK. 1974. Bunyaviruses and bunyaviridae. Intervirology 2:270-2.
56. Schmidt JRW, M. C.; Lulu, M.; Mivule, A.; Mujomba, E. 1965. Viruses isolated from mosquitoes collected in the Southern Sudan and Western Ethiopia. East African Virus Research Institute,
57. Ota WK, Watkins HM, Neri P, Schmidt ML, Schmidt JR. 1976. Arbovirus recoveries from mosquitoes collected in Gambela, Illubabor Province, Ethiopia, 1970. J Med Entomol 13:173-8.
58. Derodaniche E, Paesdeandrade A, Galindo P. 1964. Isolation of Two Antigenically Distinct Arthropod-Borne Viruses of Group C in Panama. Am J Trop Med Hyg 13:839-43.
59. Galindo P, Srihongse S. 1967. Transmission of arboviruses to hamsters by the bite of naturally infected Culex (Melanoconion) mosquitoes. Am J Trop Med Hyg 16:525-30.
60. Srihongse S, Scherer WF, Galindo P. 1967. Detection of arboviruses by sentinel hamsters during the low period of transmission. Am J Trop Med Hyg 16:519-24.
61. Karabatsos N, Buckley SM. 1967. Susceptibility of the baby-hamster kidney-cell line (BHK-21) to infection with arboviruses. Am J Trop Med Hyg 16:99-105.
62. Calisher CH, Gutierrez E, Francy DB, Alava A, Muth DJ, Lazuick JS. 1983. Identification of hitherto unrecognized arboviruses from Ecuador: members of serogroups B, C, Bunyamwera, Patois, and Minatitlan. Am J Trop Med Hyg 32:877-85.
63. Dacheux L, Berthet N, Dissard G, Holmes EC, Delmas O, Larrous F, Guigon G, Dickinson P, Faye O, Sall AA, Old IG, Kong K, Kennedy GC, Manuguerra JC, Cole ST, Caro V, Gessain A, Bourhy H. 2010. Application of broad-spectrum resequencing microarray for genotyping rhabdoviruses. J Virol 84:9557-74.
64. Begum F, Wisseman CL, Jr., Casals J. 1970. Tick-borne viruses of West Pakistan. IV. Viruses similar to or identical with, Crimean hemorrhagic fever (Congo-Semunya), Wad Medani and Pak Argas 461 isolated from ticks of the Changa Manga Forest, Lahore District, and of Hunza, Gilgit Agency, W. Pakistan. Am J Epidemiol 92:197-202.
65. Yunker CE, Clifford CM, Thomas LA, Cory J, George JE. 1972. Isolation of viruses from swallowticks, Argas cooleyi, in the southwestern United States. Acta Virol 16:415-21.
66. Clifford CM, Yunker CE, Thomas LA, Easton ER, Corwin D. 1971. Isolation of a Group B arbovirus from Ixodes uriae collected on Three Arch Rocks National Wildlife Refuge, Oregon. Am J Trop Med Hyg 20:461-8.
67. Causey OR, Shope RE, Bensabath G. 1966. Marco, Timbo, and Chaco, newly recognized arboviruses from lizards of Brazil. Am J Trop Med Hyg 15:239-43.
68. Monath TP, Cropp CB, Frazier CL, Murphy FA, Whitfield SG. 1979. Viruses isolated from reptiles: identification of three new members of the family Rhabdoviridae. Arch Virol 60:1-12.
69. Tesh RB, Travassos Da Rosa AP, Travassos Da Rosa JS. 1983. Antigenic relationship among rhabdoviruses infecting terrestrial vertebrates. J Gen Virol 64 (Pt 1):169-76.
70. Main AJ. 1978. Tindholmur and mykines: Two new Kemerovo Group Orbiviruses from the Faeroe Islands. Journal of Medical Entomology 15:11-14.
71. Schaffer PA, Scherer WF. 1972. Growth of a candidate arbovirus (Tsuruse) in Aedes aegypti mosquitoes following intrathoracic inoculation. Proc Soc Exp Biol Med 139:1298-304.

72. Bishop DHLS, R. E. 1979. Bunyaviridae, p 1-156. *In* Fraenkel-Conrat HW, R. R. (ed), Comprehensive Virology, vol 14. Plenum Press, New York.
73. Myers RM, Carey DE, Reuben R, Jesudass ES, Shope RE. 1971. Vellore virus: a recently recognized agent of the Palyam group of arboviruses. Indian J Med Res 59:1209-13.
74. Elbel REC, G.T.; Calisher, C.H. 1977. Arbovirus isolations from southwestern Utah and northwestern Arizona insects, 1972–1975. Mosquito News 37:497-507.
75. Pauvolid-Correa A, Campos Z, Soares R, Nogueira RMR, Komar N. 2017. Neutralizing antibodies for orthobunyaviruses in Pantanal, Brazil. PLoS Negl Trop Dis 11:e0006014.
76. Gerrard SR, Li L, Barrett AD, Nichol ST. 2004. Ngari virus is a Bunyamwera virus reassortant that can be associated with large outbreaks of hemorrhagic fever in Africa. J Virol 78:8922-6.
77. Calisher CH, Sabattini MS, Monath TP, Wolff KL. 1988. Cross-neutralization tests among Cache Valley virus isolates revealing the existence of multiple subtypes. Am J Trop Med Hyg 39:202-5.
78. Lambert AJ, Lanciotti RS. 2008. Molecular characterization of medically important viruses of the genus Orthobunyavirus. J Gen Virol 89:2580-2585.
79. Yunker CE, Clifford CM, Keirans JE, Thomas LA, Cory J. 1973. Tickborne viruses in western North America. II. Yaquina head, a new arbovirus of the Kemerovo group isolated from Ixodes uriae. J Med Entomol 10:264-9.
80. Teufel F, Almagro Armenteros JJ, Johansen AR, Gislason MH, Pihl SI, Tsirigos KD, Winther O, Brunak S, von Heijne G, Nielsen H. 2022. SignalP 6.0 predicts all five types of signal peptides using protein language models. Nat Biotechnol 40:1023-1025.
81. Martin DP, Varsani A, Roumagnac P, Botha G, Maslamoney S, Schwab T, Kelz Z, Kumar V, Murrell B. 2021. RDP5: a computer program for analyzing recombination in, and removing signals of recombination from, nucleotide sequence datasets. Virus Evol 7:veaa087.
